# Delineating the Role of the *msaABCR* Operon in Staphylococcal Overflow Metabolism

**DOI:** 10.3389/fmicb.2022.914512

**Published:** 2022-06-03

**Authors:** Bibek G C, Gyan S. Sahukhal, Mohamed O. Elasri

**Affiliations:** ^1^Center for Molecular and Cellular Biosciences, The University of Southern Mississippi, Hattiesburg, MS, United States; ^2^Department of Microbiology and Immunology, University of Arkansas for Medical Sciences, Little Rock, AR, United States

**Keywords:** *Staphylococcus aureus*, *msaABCR* operon, overflow metabolism, pyruvate catabolism, *cidR* regulon, programmed cell death, biofilm formation

## Abstract

*Staphylococcus aureus* is an important human pathogen that can infect almost every organ system, resulting in a high incidence of morbidity and mortality. The *msaABCR* operon is an important regulator of several staphylococcal phenotypes, including biofilm development, cell wall crosslinking, antibiotic resistance, oxidative stress, and acute and chronic implant-associated osteomyelitis. Our previous study showed that, by modulating murein hydrolase activity, the *msaABCR* operon negatively regulates the proteases that govern cell death. Here, we report further elucidation of the mechanism of cell death, which is regulated by the *msaABCR* operon at the molecular level in the USA300 LAC strain. We showed that deletion of *msaABCR* enhances weak-acid-dependent cell death, because, in the biofilm microenvironment, this mutant strain consumes glucose and produces acetate and acetoin at higher rates than wild-type USA300 LAC strain. We proposed the increased intracellular acidification leads to increased cell death. MsaB, a dual-function transcription factor and RNA chaperone, is a negative regulator of the *cidR* regulon, which has been shown to play an important role in overflow metabolism and programmed cell death during biofilm development in *S. aureus*. We found that MsaB binds directly to the *cidR* promoter, which represses expression of the *cidR* regulon and prevents transcription of the *cidABC* and *alsSD* operons. In addition, we observed that pyruvate induced expression of the *msaABCR* operon (MsaB). The results reported here have enabled us to decipher the role of the *msaABCR* operon in staphylococcal metabolic adaption during biofilm development.

## Introduction

*Staphylococcus aureus* is a human pathogen that significantly impacts both community and health care settings, causing a wide spectrum of community-acquired and nosocomial infections, respectively. A growing concern for treatment of *S. aureus* is the increase in its formation of biofilms within host tissues and on the surfaces of implanted medical devices. These biofilms confer protection against antibiotics, stimulate resistance against clearance by the host immune response, and support spread from the infection site (Davis et al., 2008; Otto, 2018), leading to chronic wound infections and systemic diseases, such as bacteremia, osteomyelitis, and endocarditis (Petti and Fowler, 2002; Tong et al., 2015; Dayan et al., 2016). Biofilms are structured bacterial communities in which bacteria stick together or to a surface by self-produced, sticky polymeric molecules, including polysaccharides (e.g., polysaccharide intercellular adhesin), extracellular DNA (eDNA), and proteins (Otto, 2018), as well as others. It is expected that elucidating the mechanisms of formation and maintenance of bacterial biofilms will help identify new strategies for inhibiting their production.

Programmed cell death (PCD) is a phenomenon by which some cells undergo a suicidal mechanism for the benefit of the whole organism and is well defined in eukaryotes (Rice and Bayles, 2003; Bayles, 2007). The concept of PCD has been extended to prokaryotes that develop multicellular communities, such as bacterial biofilms, with a particular focus on production of eDNA and pronounced death and lysis during bacterial biofilm development (Rice and Bayles, 2003; Bayles, 2007; Moormeier and Bayles, 2017). The main purpose of bacterial PCD in this context is hypothesized to be the release of genomic DNA, proteins, and polysaccharides that serve as constituents of the biofilm matrix. However, this process is a finely balanced phenomenon, as several bacterial mutants defective in lysis and the release of eDNA are poor biofilm formers (Rice et al., 2003, 2005; Patton et al., 2005; Yang et al., 2006; Bayles, 2007; Thomas et al., 2014; Chaudhari et al., 2016; Windham et al., 2016). The LysR-type transcriptional regulator, CidR, is a transcriptional activator of the *cidABC*, *lrgAB*, and *alsSD operons* (i.e.*, cidR regulon*) and has been shown to play an important role in autolysis and cell death during biofilm formation (Rice et al., 2003, 2005; Patton et al., 2005; Yang et al., 2006; Thomas et al., 2014; Chaudhari et al., 2016; Windham et al., 2016; Sadykov et al., 2019).

In addition to the *cidA*–*lrgA* holin–antiholin system, other genes, such as *cidC* and *alsSD,* regulate PCD by modulating overflow metabolism in *S. aureus* (Patton et al., 2005; Yang et al., 2006; Thomas et al., 2014; Chaudhari et al., 2016; Sadykov et al., 2019). When grown under aerobic conditions, most of the carbon (including glucose) is directed to substrate-level phosphorylation that is catalyzed by the Pta–AckA pathway, which is important for yielding secondary energy, producing acetate when activity of the TCA cycle is limited by repression of carbon catabolites (known as the Crabtree effect; Sadykov et al., 2013; Marshall et al., 2016). When glucose is completely exhausted in the medium, *S. aureus* generates the energy required for classic diauxic growth by reusing acetate that was excreted *via* the TCA cycle (Somerville et al., 2002, 2003; Thomas et al., 2014). Overflow metabolism is a wasteful strategy for catabolizing excess nutrients through incomplete oxidation, even under aerobic conditions, because it produces fewer energy metabolites per glucose molecule than the energy-efficient respiration pathway (Thomas et al., 2014). *S. aureus* overflow metabolism involves catabolism of excess pyruvate that is produced by increased glycolytic flux that exceeds its TCA flux. This results in the formation of acetate *via* the AckA–Pta and CidC pathways, of acetoin *via* the AlsSD pathway, and of 2,3-butanediol *via* the ButA pathway (Patton et al., 2005; Yang et al., 2006; Sadykov et al., 2013; Thomas et al., 2014; Chaudhari et al., 2016; Marshall et al., 2016; Zhang et al., 2017).

Two genes of the CidR regulon—*cidC* (encoding pyruvate:menaquinone oxidoreductase) and *alsSD* (encoding α-acetolactate synthetase/decarboxylase)—have enzymatic products that use the same substrate—pyruvate—and produce acetate and acetoin, respectively (Patton et al., 2005; Yang et al., 2006; Thomas et al., 2014; Zhang et al., 2017). Acetate and acetoin metabolites affect cell death antithetically, suggesting an intimate relationship between pyruvate metabolism and cell death. *cidC*-encoded pyruvate oxidase promotes cell death during stationary phase by increasing acidification of the growth medium *via* production of acetate (Thomas et al., 2014). The *alsSD* operon encodes α-acetolactate synthase (AlsS) and α-acetolactate decarboxylase (AlsD), which catabolize pyruvate to acetoin, which can be processed by acetoin reductase (ButA) to create 2,3-butanediol (Yang et al., 2006; Thomas et al., 2014; Chaudhari et al., 2016). The synthesis of acetoin and 2,3-butanediol (both neutral metabolites) redirects pyruvate catabolism away from the *cidC* pathway and actively consumes protons, promoting a neutral environment (Thomas et al., 2014).

While CidR is the main transcriptional activator of the *cidABC* and *alsSD* operons, other regulators, such as SrrAB and CcpA, also are involved in the complex regulatory network of these operons (Patton et al., 2005, 2006; Yang et al., 2006; Chaudhari et al., 2016; Windham et al., 2016; Sadykov et al., 2019). The SrrAB two-component system, which responds to nitric oxide stress and oxygen availability, appears to repress expression of the *cidABC* operon (Kinkel et al., 2013; Windham et al., 2016). Furthermore, in agreement with the facts that excess glucose is needed to induce expression of the *cidABC* operon and that CcpA is a master regulator of carbohydrate metabolism, both CcpA and CidR are required to fully induce expression of *cidABC* and *alsSD* (Seidl et al., 2009; Sadykov et al., 2011, 2019).

The *msaABCR* operon in *S. aureus* previously was found to regulate expression of global regulator *sarA*, as well as biofilm development, virulence, antibiotic resistance, persistence, and chronic implant-associated osteomyelitis (Sahukhal and Elasri, 2014; Samanta and Elasri, 2014; Sahukhal et al., 2015, 2017, 2020; Batte et al., 2018; GC et al., 2019; Pandey et al., 2019; Rom et al., 2020). The four-gene operon is composed of *msaA*, *msaB*, *msaC*, and the antisense RNA *msaR* (Sahukhal and Elasri, 2014). *msaA, msaC*, and *msaR* are noncoding RNAs and are thought to regulate expression of MsaB. *msaB* is the only protein-coding gene of the *msaABCR* operon; it encodes the MsaB protein, which acts as a transcription factor and as an RNA chaperone (Sahukhal and Elasri, 2014; Batte et al., 2016, 2018; Pandey et al., 2019). Studies from our laboratory have shown that deletion of the *msaABCR* operon results in decreased minimum inhibitory concentration for antibiotics that target the cell wall (e.g., vancomycin and certain β-lactams) in vancomycin-intermediate *S. aureus* (VISA) strains (Mu50, HIP6297, and LIM2) and in a methicillin-resistant *S. aureus* (MRSA) strain (USA300 LAC; Samanta and Elasri, 2014; Sahukhal et al., 2017; GC et al., 2019). We also showed that deletion of the *msaABCR* operon in *S. aureus* cells results in a significant decrease in biofilm thickness and in a significant increase in cell death, relative to the wild-type strain (Sahukhal et al., 2015). Notably, the biofilm defect in the ∆*msaABCR* mutant resulted from uncontrolled cell death (Sahukhal et al., 2015). Furthermore, we demonstrated that the *msaABCR* operon represses expression of genes encoding four extracellular proteases (*Aur*, *Scp*, *Ssp.*, and *Spl*) in different growth phases (Sahukhal and Elasri, 2014; Sahukhal et al., 2015). Serine protease Ssp., the cysteine proteases, and other proteases are involved in processing AtlA, a major murein hydrolase, thereby regulating murein hydrolase activity and cell death during biofilm development (Rice et al., 2001; Thomas et al., 2008; Chen et al., 2013; Sahukhal et al., 2015).

These findings have led to the present study, in which we further elucidated the interrelation between the *msaABCR* operon (MsaB) and the CidR regulon in regulating pyruvate homeostasis, overflow metabolism, and programmed cell death during biofilm development.

## Materials and Methods

### Bacteria and Growth Conditions

Experiments were conducted with USA300 LAC, a clinically significant community-acquired MRSA strain. The detailed lists of all the strains and mutant strains used in this study are listed in Supplementary Table 1. The allelic replacement method was used to generate *msaB* and *msaABCR-*deletion mutants in USA300 LAC (Bae and Schneewind, 2006; Elbarasi, 2014; Samanta and Elasri, 2014). For trans-complementation, the *msaABCR* region was cloned into pCN34, a low-copy vector that was modified to replace the kanamycin selectable marker with a chloramphenicol-resistance marker, as described previously (Samanta and Elasri, 2014). The *cidC:Tn*, *CidR:Tn*, ∆*msaABCR*/*cidC:Tn*, and ∆*msaABCR/cidC:Tn* mutants were created by transducing, *via* bacteriophage Φ11, *cidC:Tn* and *cidR:Tn* (*bursa aurealis* transposon mutants, obtained from Nebraska Transposon Mutant Library) to USA300 LAC wild-type strain and ∆*msaABCR* mutant strains. *S. aureus* strains were grown in tryptic soy agar (TSA) or tryptic soy broth (TSB). Overnight bacterial cultures were prepared by inoculating cells from frozen culture stocks into culture tubes containing 5 ml of freshly prepared TSB or Mueller–Hinton broth and incubating at 37°C with continuous shaking (225 rpm). The overnight cultures were diluted 1:10 in fresh medium, incubated for 2 h, and then normalized to 0.05 optical density at 600 nm (OD_600_) for use as the starting cultures for experiments.

### Biofilm Assays

Biofilm assays were performed according to the original protocol outlined by Sambanthamoorthy et al., with modifications added by Sahukhal and Elasri (Sambanthamoorthy et al., 2008; Sahukhal and Elasri, 2014). First, we pre-coated each well of the microtiter plates with 20% human plasma and incubated the plates overnight at 4°C. Next, each well was inoculated with 2 ml of starter culture (prepared as described above, “Bacteria and growth conditions”). The biofilm medium for this assay was prepared by mixing TSB with 3% NaCl and 0.25% glucose; for excess glucose conditions, an additional 0.5% glucose was added to the appropriate wells. We also added vancomycin (0.2 μg/ml) when necessary. The microtiter plates were incubated at 37°C for 24 h with shaking at 150 rpm. After 24 h, we washed each well with sterile phosphate-buffered saline (PBS), stained the resulting biofilms with crystal violet dye, and eluted with 5% acetic acid. To measure the amount of biofilm, we used a spectrophotometer to quantitate absorbance at 595 nm. The assays were performed in experimental duplicates for USA300 LAC (wild type), ∆*msaABCR* mutant, and complementation strains; biofilm assays were performed three times. Biofilm formation values were calculated as the percent activity relative to USA300 LAC (wild type), which was set as 100%; mean values and standard deviations of each growth condition then were calculated.

### Growth Curves and CFU Count

To determine the effects of excess glucose on cell death, the survival of different strains was monitored for 5 days (120 h). Wild type, ∆*msaABCR* mutant, and complementation strains of USA300 LAC were grown aerobically in TSB with 50 mM glucose (TSB-50 mM glucose) and, when indicated, buffered with morpholinepropanesulfonic acid (MOPS) buffer (50 mM, pH 7.3). Cultures were incubated for 120 h at 37°C with shaking at 220 rpm, and samples were collected at 24-h intervals. To measure the colony-forming units (CFUs) of each sample, cultures were serially diluted up to the appropriate dilutions, and 100-μl aliquots were placed on TSA plates, which then were incubated overnight at 37°C. To verify the consistency of these results, cell death assays for each sample were performed in triplicate and were repeated at least three times. The CFUs for each sample were averaged to obtain mean values, and the standard deviation was calculated.

### Metabolite Analyses

For these analyses, bacterial growth was allowed to proceed (at 37°C and 225 rpm) in flasks containing TSB-50 mM glucose at a 1:10 flask-to-volume ratio. Metabolite excretion profiles were determined from culture supernatants that were harvested at the indicated times and under the indicated growth conditions after incubation. Glucose and acetate were measured with commercial kits (R-Biopharm), according to the manufacturer’s instructions.

Acetoin assays were performed as previously described (Nicholson, 2008). Briefly, 300 μl of supernatant was mixed with 210 μl of 0.5% creatine, 300 μl of 5% α-napthol, and 300 μl of 40% KOH. Each sample was incubated for 15–30 min. OD_560_ was measured and used to determine the concentration of acetoin.

### RNA Extraction, Reverse Transcription, and Quantitative Reverse-Transcription PCR

Starting cultures were grown in the specified growth medium for 5 h, and the cells were harvested by centrifugation. The bacterial pellet was treated with RNAprotect bacterial reagent (Qiagen), and the total RNA was extracted as described previously (Samanta and Elasri, 2014; Sahukhal et al., 2015). Relative gene expression was quantified with real-time PCR analysis of cDNA prepared from the total RNA samples. All experiments used housekeeping gene *gyrB* as an internal control (Goerke et al., 2000; Samanta and Elasri, 2014; Sahukhal et al., 2015). The relative fold-change in gene expression was calculated with the ∆CT method. All primers used for qRT-PCR are listed in Supplementary Table 2.

### Expression and Purification of MsaB

MsaB protein was cloned into pCN51, an inducible expression vector that, when induced, produces the protein with 6 × His (MsaB_his_) at the C-terminus. The construct was transformed into *E. coli* strain DH5α, and the plasmids isolated from these cells were used to transduce competent cells of the restriction-deficient *S. aureus* RN4220 strain. The plasmid then was moved to the USA300 LAC ∆*msaABCR* mutant through generalized transduction with bacteriophage ϕ11, as described previously (Pandey et al., 2019). To induce expression of the 6 × His–MsaB fusion protein (i.e., MsaB_his_) in the ∆*msaABCR* mutant of the USA300 LAC strain, 20 μM cadmium chloride (CdCl_2_) was added to cell cultures during exponential growth phase; cell cultures were incubated an additional 4 h with shaking. The cells then were pelleted, resuspended in PBS (pH 7.4) with a protease inhibitor cocktail, and lysed by bead beating followed by sonication. The cell lysate was centrifuged at 10,000×*g* for 30 min to remove cell debris, and MsaB_his_ was purified from the clear lysate with a nickel column (HisPur Ni-nitrilotriacetic acid [Ni-NTA] resin; Thermo Scientific).

### Electrophoretic Mobility Shift Assay

The ability of MsaB to bind the *cidR* promoter was determined according to electrophoretic mobility shift, as described previously (Pandey et al., 2019). Briefly, the 5′-biotinylated P*cidR*-Electrophoretic Mobility Shift assay (EMSA) forward primers and reverse primers were used to amplify the promoter region. The PCR product (250 μl) was loaded onto a 2% agarose gel and separated by electrophoresis, and the corresponding P*cidR* fragment was extracted from the gel (Wizard SV Gel and PCR Cleanup System, Promega). The concentration and quality of the purified DNA product were determined with a NanoDrop spectrophotometer (Thermo Scientific). To study the ability MsaB to bind to the *CidABC* and *alsSD* promoters, the 5′-biotinylated duplex oligonucleotide sequence of the promoter regions of these genes was used, as in previous studies (Sadykov et al., 2019). For EMSA experiments, the LightShift Chemiluminescent EMSA kit (Pierce) was used according the manufacturer’s protocol. The binding reaction mixture (20 μl) contained ultrapure water, 1X binding buffer, 50 ng μl^− 1^ poly (dI-dC), 2.5% (vol/vol) glycerol, 5 mM MgCl_2_, and 5′-biotin-labeled DNA probe; increasing concentrations of MsaB_his_ protein and unlabeled specific probe, when required, were added. The reaction mixture was incubated at room temperature for 20 min and separated by electrophoresis (1 h, 100 V) in a pre-run 5% Tris-borate-EDTA (TBE) gel. The samples in the gel were then transferred to a nylon membrane (1 h, 4°C), cross-linked in a UV crosslinker, and processed for detection. The blots were developed and visualized with the kit detection module, according to the manufacturer’s protocol, and were imaged with the ChemiDoc system (Bio-Rad).

### Murein Hydrolase Assay

Starter culture was incubated for 16 h at 37°C with shaking at 250 rpm. The culture supernatants were collected by centrifugation and were concentrated (Centricon-3 concentrator; Millipore) approximately 10-fold. Protein concentrations of the extracellular supernatants were determined with the BCA protein assay kit (Life Technologies), according to the manufacturer’s recommendations. Quantitative cell wall hydrolysis assays were performed as described previously (Sahukhal et al., 2015).

### Western Blotting of MsaB_his_

To quantify the amount of MsaB_his_ produced under different growth conditions, Western blotting was performed with whole-cell lysates, as described previously (Batte et al., 2016). The USA300 LAC *msaAB_his_CR* complementation strain was used for this study. It was generated by introducing the pCN34–*msaAB_his_CR* operon construct (with His-tagged MsaB at the 5′ end) into USA300 LAC ∆*msaABCR*. The USA300 LAC *msaAB_his_CR* complementation cells were grown in TSB without glucose, TSB-50 mM glucose, and TSB without glucose supplemented with 2% pyruvate. Cells were grown for 5 h, pelleted by centrifugation, washed with PBS, resuspended in lysis buffer (PBS with a protease inhibitor), and lysed with a Fastprep instrument with glass beads. The clear supernatants were obtained after centrifugation of the whole-cell lysates to remove debris. Proteins from different growth conditions were quantified with the BCA method (Pierce BCA protein assay kit; Life Technologies), and 25 μg of proteins were separated with SDS-polyacrylamide gel electrophoresis. Proteins in the gels were transferred to polyvinylidene difluoride (PVDF) membrane by blotting, blots were blocked wit 5% non-fat milk, and MsaB_his_ was detected in the blots with an anti-His antibody and a peroxidase-conjugated secondary antibody.

### Statistical Analysis

All statistical analyses to test for significant differences in this study were done with OriginPro software (Origin Lab). A statistical significance level of *p* < 0.05 was used as the cutoff for significance in performing statistical analyses between the strains. Student’s *t*-test (unpaired) or one-way analysis of variance (ANOVA) were used to compare the results from wild-type strains with those from mutant or complementation strains (^*^p < 0.05, ^**^p < 0.005, ^***^p < 0.0005).

## Results

### The *msaABCR* Operon Represses Acetate-Mediated Cell Death During Biofilm Formation

**Figure 1:**
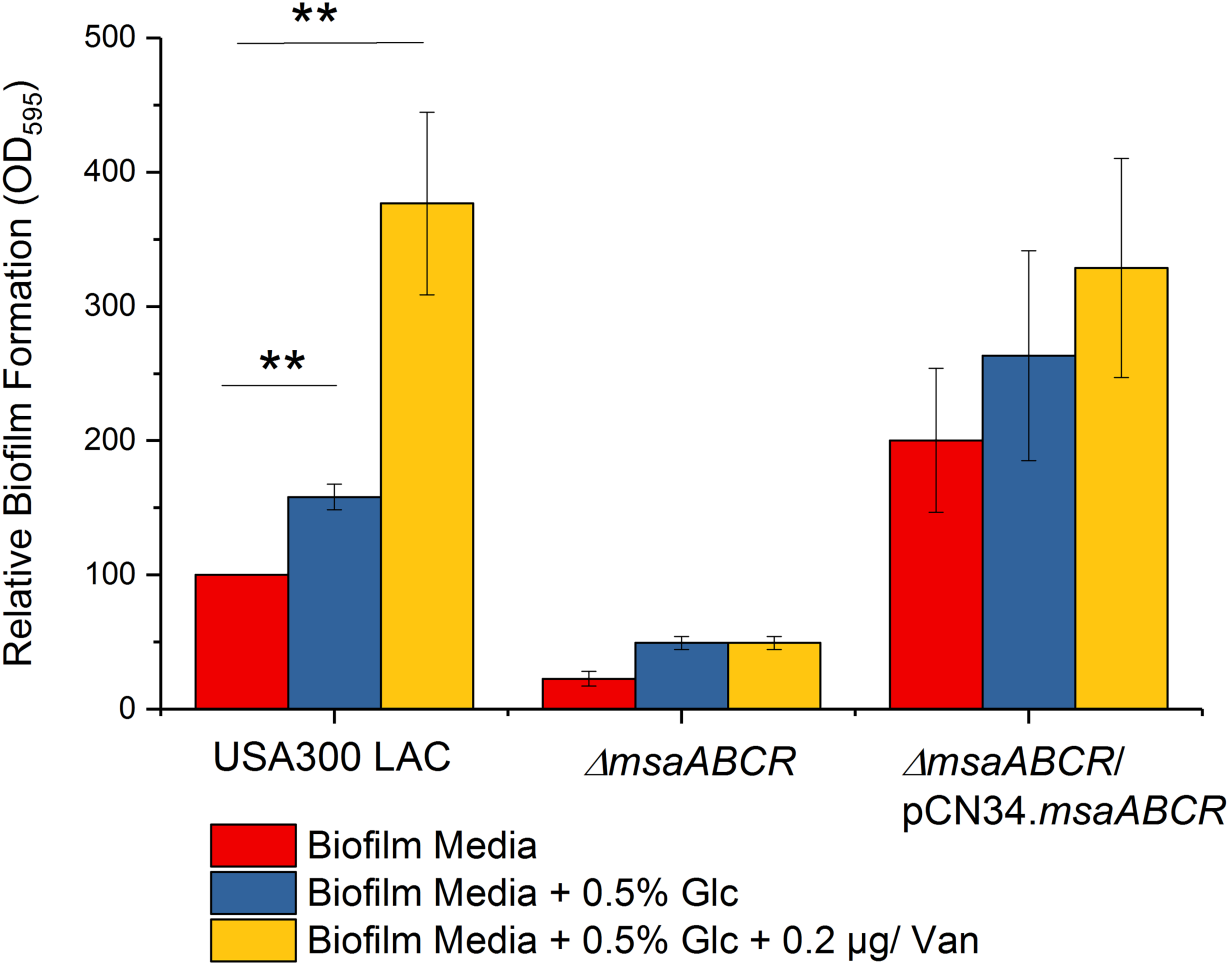
Biofilm formation by the USA300 LAC (wild type), ∆*msaABCR* mutant, and complementation strains. Biofilm formation measured in biofilm medium, biofilm medium supplemented with excess glucose (0.5%), and biofilm medium supplemented with excess glucose (0.5%) and a sub-inhibitory concentration of vancomycin (0.2 μg/ml). Values are shown as the percent activity relative to the wild-type USA300 LAC, which was set at 100%. Each vertical bar represents the mean of three independent experiments. Error bars represent the standard error (SE). One-way ANOVA was used to compare the results for wild-type, ∆*msaABCR* mutant, and complementation strains in different growth media (^**^p < 0.005).

We performed biofilm assays under conditions of excess glucose (0.5%) and/or a sub-inhibitory concentration of vancomycin in the USA300 LAC (wild type), ∆*msaABCR* mutant, and complementation strains. The USA300 LAC strain showed significantly increased biofilm production under the supplementation conditions tested (*p* < 0.005); however, under these same conditions, biofilm production was not induced to significant level in the ∆*msaABCR* mutant (Figure 1). These observations suggest that the *msaABCR* operon plays an important role in biofilm formation in the presence of excess glucose, and/or subinhibitory concentration of vancomycin.

**Figure 2:**
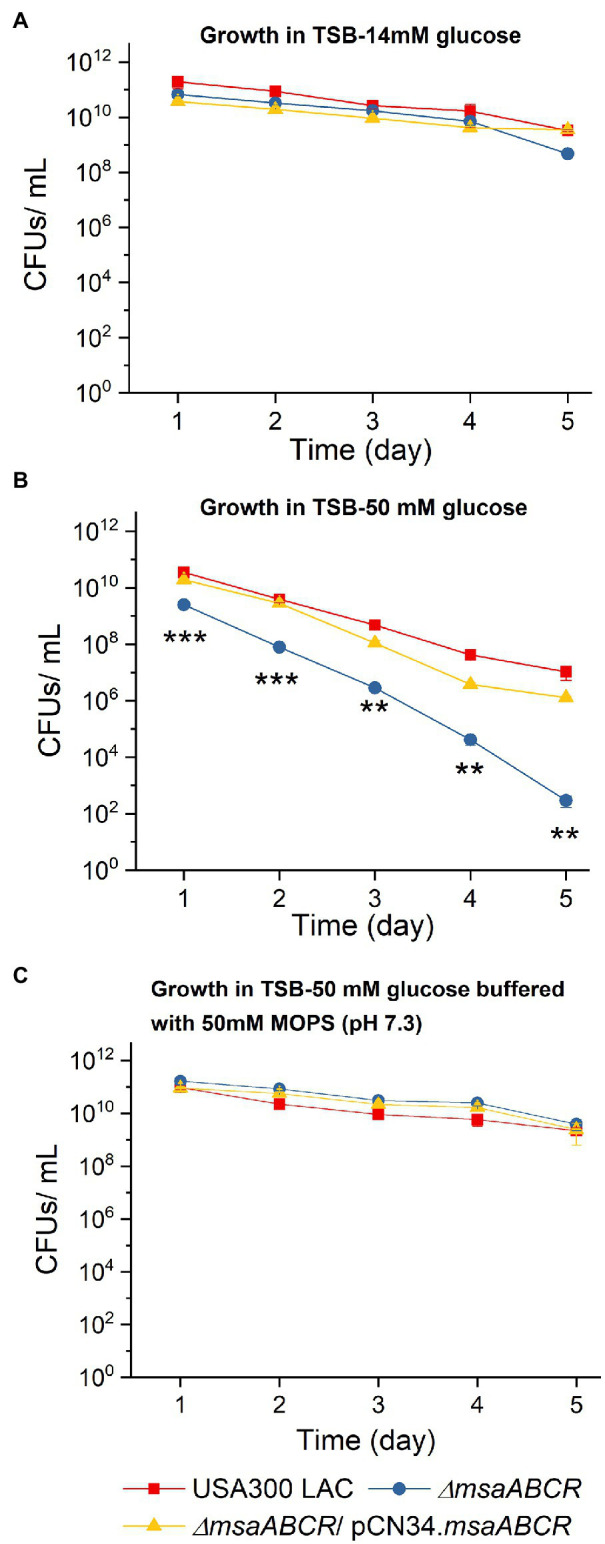
Survival of the ∆*msaABCR* mutant in stationary phase. Cell viability of *Staphylococcus aureus* USA300 LAC (wild type), ∆*msaABCR* mutant, and complementation strain was monitored every 24 h over a period of 5 days in TSB plus 14 mM glucose **(A)**, TSB-50 mM glucose **(B)**, and TSB-50 mM glucose buffered with MOPS (pH 7.3; **C**). Each data point represents the mean of three independent experiments. Error bars represent the SE. One-way ANOVA was used to compare the results for wild type with those of its mutant and complementation strains (^**^p < 0.005, ^***^p < 0.0005).

Previously, we showed that a phenotype of increased cell death was associated with the ∆*msaABCR* mutation under conditions for both planktonic and biofilm growth (Samanta and Elasri, 2014; Sahukhal et al., 2015; GC et al., 2019). Other studies reported that aerobic growth of *S*. *aureus* in TSB-50 mM glucose impairs stationary-phase survival of *S*. *aureus,* due to accumulation of acetate derived from the incomplete catabolism of glucose, thus potentiating cell death (Thomas et al., 2014; Augagneur et al., 2020). Therefore, we monitored survival of the USA300 LAC (wild type), ∆*msaABCR* mutant, and complementation strains during stationary phase when cultured in TSB supplemented with 14 or 50 mM glucose. In TSB with 14 mM supplemental glucose, all test strains, including the ∆*msaABCR* mutant, demonstrated uncompromised survival during stationary phase over a period of 5 days (Figure 2A). However, in TSB-50 mM glucose, survival of the ∆*msaABCR* mutant, relative to that of the USA300 LAC and complementation strains, significantly decreased over a period of 5 days (Figure 2B). Based on these results, we hypothesized that intracellular acidification might be responsible for the reduced survival seen in the ∆*msaABCR* mutant. To assess whether the reduced cell survival of the ∆*msaABCR* mutant grown in excess glucose was indeed caused by intracellular acidification, we performed a cell viability assay in TSB-50 mM glucose and buffered with 50 mM morpholinepropanesulfonic acid (MOPS, pH 7.3). Under these conditions, survival of the ∆*msaABCR* mutant improved and reverted to the level of USA300 LAC (Figure 2C). Together, these results suggest that the observed increase in cell death of the ∆*msaABCR* mutant strain is due to cellular acidification.

**Figure 3:**
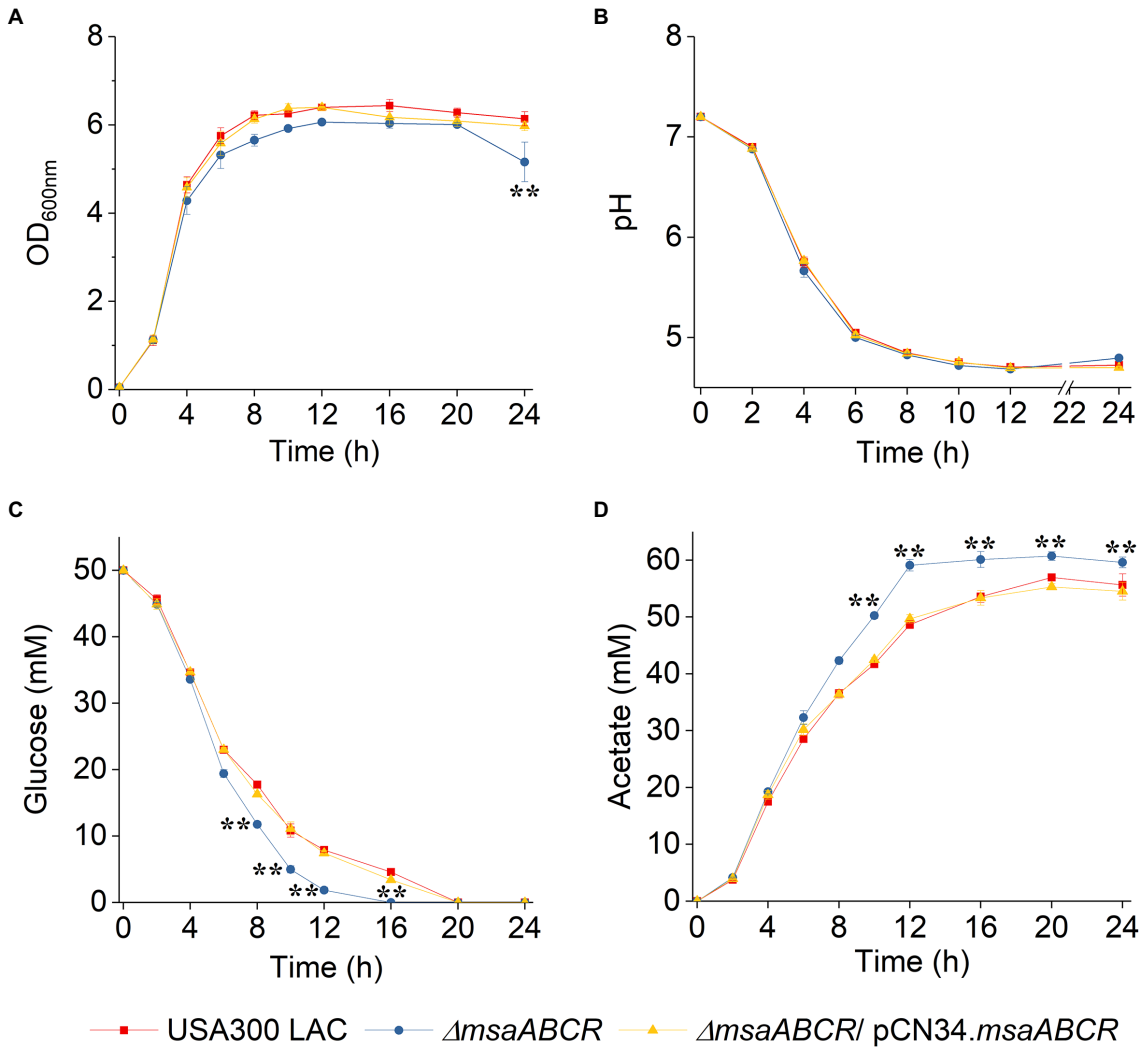
Growth curve, pH kinetics, glucose kinetics, and acetate kinetics of the USA300 LAC (wild type), ∆*msaABCR* mutant, and complementation strains grown aerobically in TSB-50 mM glucose. The OD_600nm_ **(A)** and pH **(B)** of the culture medium were determined at the indicated times. Temporal depletion of glucose from **(C)** or accumulation of acetic acid in **(D)** the culture media of the indicated strains. Glucose and acetate concentrations in culture supernatants were measured at the indicated from 0 to 24 h of growth in TSB-50 mM glucose. Each data point represents the mean of three independent experiments. Error bars represent the SE. One-way ANOVA was used to compare the results for wild type with those of the mutant and complementation strains (^**^p < 0.005).

Studies have shown that when *S. aureus* is grown aerobically under excess glucose conditions, pyruvate is catabolized to acetate *via* the AckA–Pta and CidC pathways (Patton et al., 2005; Yang et al., 2006; Thomas et al., 2014; Chaudhari et al., 2016; Zhang et al., 2017). To examine how the *msaABCR* operon is involved in glucose catabolism to acetate, we measured glucose utilization and acetate production in all three test strains by growing the cells for 24 h in TSB-50 mM glucose. OD_600_ of all test strains was similar for the first 12 h, but OD_600_ the ∆*msaABCR* mutant was significantly reduced after 24 h of growth (Figure 3A), most likely due to increased lysis of ∆*msaABCR* mutant cells. When we measured the pH of the supernatants collected from cell cultures, we observed that all strains underwent similar changes in pH over 24 h (Figure 3B). Measurements of glucose and acetate kinetics showed that the ∆*msaABCR* mutant consumed glucose and produced acetate significantly faster after late exponential growth phase (4 h) than the USA300 LAC (wild type) and complementation strains (Figures 3C,D). The **∆**msaABCR mutant consumed the most glucose before 12 h of growth and had minimal glucose (~2 mM) remaining in the medium at 12 h; however, the wild type and complementation strains maintained ~4 mM glucose in the medium, even after 16 h of growth (Figure 3C). Acetate levels for all the strains remained almost constant from 16 to 24 h, suggesting that none of the strains could reuse acetate when grown in excess glucose, even after glucose was depleted from the culture medium (Figure 3D). Therefore, these strains did not undergo classic diauxic growth in an acidic growth environment (Figure 3A).

**Figure 4:**
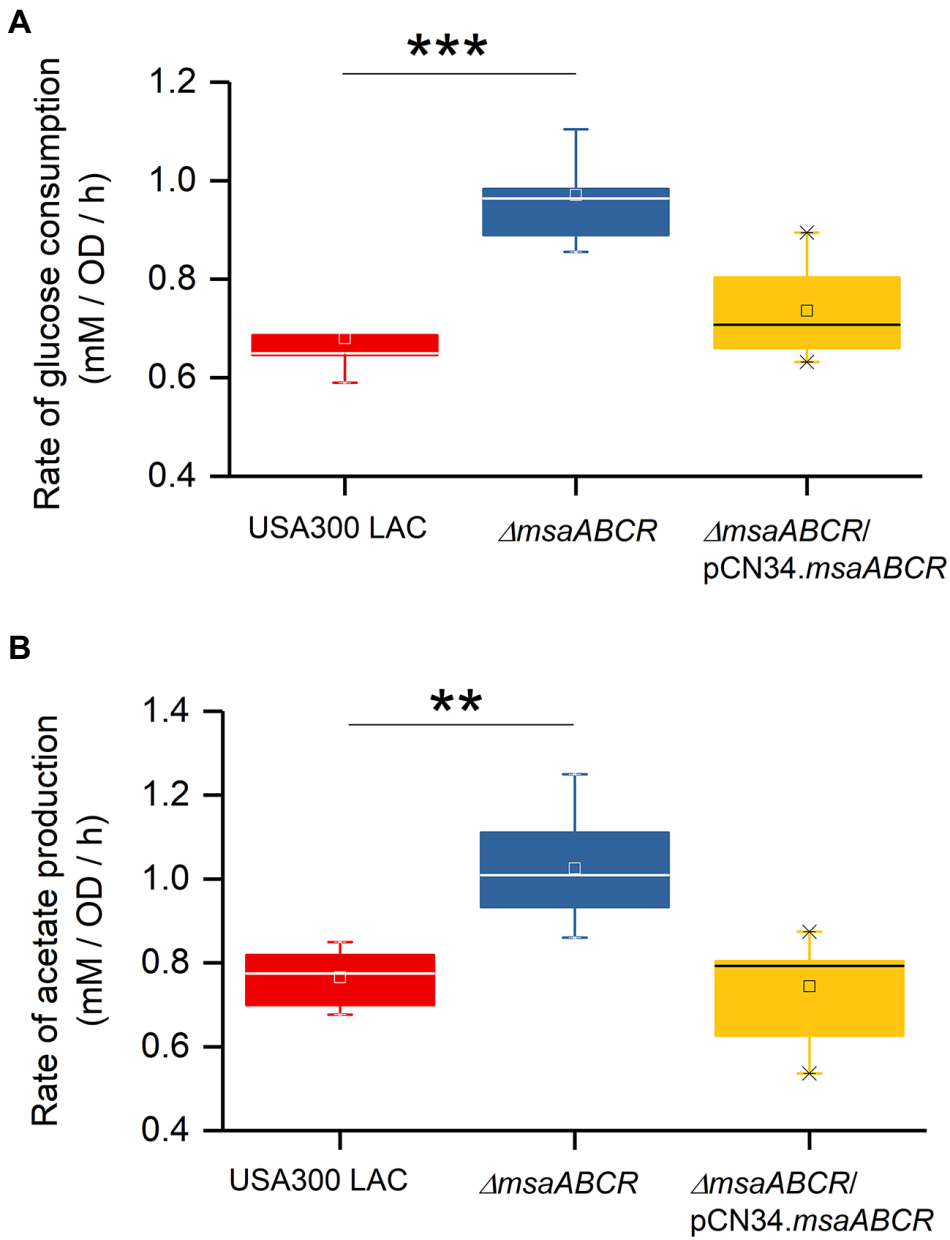
Rate of glucose consumption and production of acetate under TSB-50 mM growth conditions. The rate of glucose consumption was measured as the concentration of glucose (mM) consumed from 4 to 8 h of growth per OD_600_ at the 8-h time point per unit time (h; **A**). The rate of acetate production was measured as the concentration of acetate (mM) produced from 4 to 8 h of growth per OD_600_ at the 8-h time point per unit time (h) in *Staphylococcus aureus* USA300 LAC (wild type) and the ∆*msaABCR* mutant strains **(B)**. Glucose and acetate concentration in culture supernatants were measured at the indicated growth times in TSB-50 mM glucose. The box indicates Q1 and Q3 values; the solid line represents Q2. One way ANOVA was used to compare the results for wild type with those of the mutant and complementation strains (^**^p < 0.005, ^***^p < 0.0005).

Because we observed the effects of msaABCR deletion after late exponential phase (after 4 h) and the growth medium pH reached ~4.8 at 8 h (Figure 3B), the period of 4–8 h was chosen for measuring the rates of glucose consumption and acetate production. These rates were measured as the difference in acetate or glucose levels in the growth medium from 4 to 8 h of growth per unit time per OD_600_ at 8 h. When we measured the rate of glucose consumption and acetate production in this way, we observed that the ∆*msaABCR* mutant had a significantly (*p* < 0.0005) higher rate of glucose consumption (Figure 4A) and a significantly (*p* < 0.005) higher rate of acetate production (Figure 4B) than the USA300 LAC (wild type) and complementation strains. In an acidic environment (pH ~ 4.76 = pKa of acetate), the equilibrium between acetate and acetic acid shifts toward the neutral form—acetic acid. Neutral acetic acid then can pass freely through the cell membrane, which is thought to be the cause of intracellular acidification (Thomas et al., 2014). Thus, increased acetate production in the ∆*msaABCR* mutant under lower pH conditions (close to pH 4.8), suggests an increase in neutral acetic acid, which can diffuse across the membrane and cause intracellular acidification in the mutant compared with the USA300 LAC (wild type) and complementation strains. Thus, we hypothesized that the decreased viability of the ∆*msaABCR* mutant during stationary phase (24–120 h) in TSB-50 mM glucose (Figure 2B) is due to increased intracellular acidification and its pleiotropic effects.

**Figure 5:**
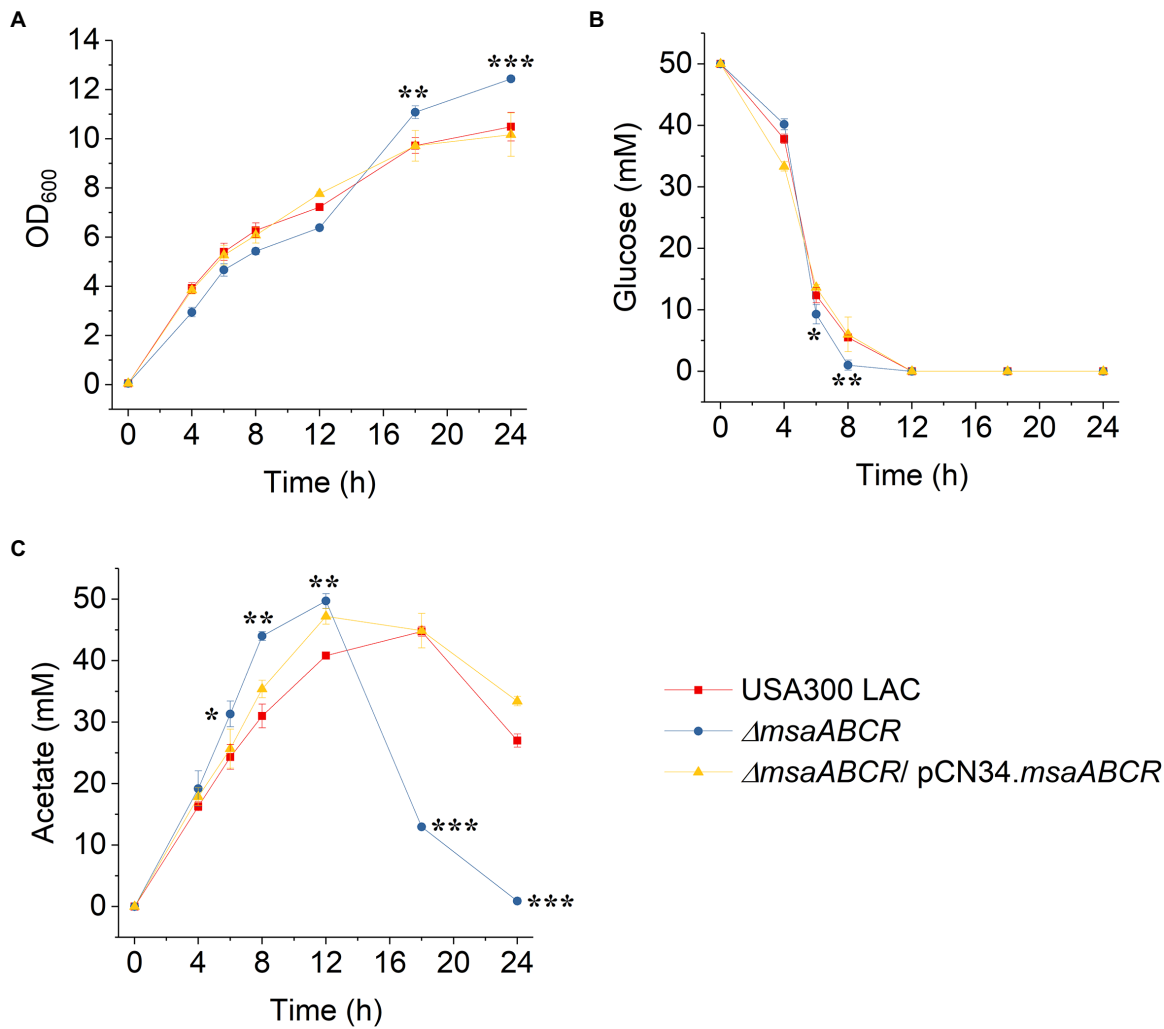
Growth and kinetics of glucose and acetate for the USA300 LAC (wild type), ∆*msaABCR* mutant, and complementation strains grown aerobically in TSB-50 mM glucose buffered with MOPS (50 mM, pH 7.3). The OD_600_ of the culture medium was determined at the indicated times **(A)**. Temporal depletion of glucose from **(B)** or accumulation of acetic acid and reuse in **(C)** the culture media of the indicated strains. Glucose and acetate concentrations in culture supernatants were measured at the indicated time of culture from 0 to 24 h of growth in TSB-50 mM glucose buffered with 50 mM MOPS, pH 7.3. Each data point represents the mean of three independent experiments. Error bars represent the SE. One-way ANOVA was used to compare the results for wild type with those of the mutant and complementation strains (^*^p < 0.05, ^**^p < 0.005, ^***^p < 0.0005).

**Figure 6:**
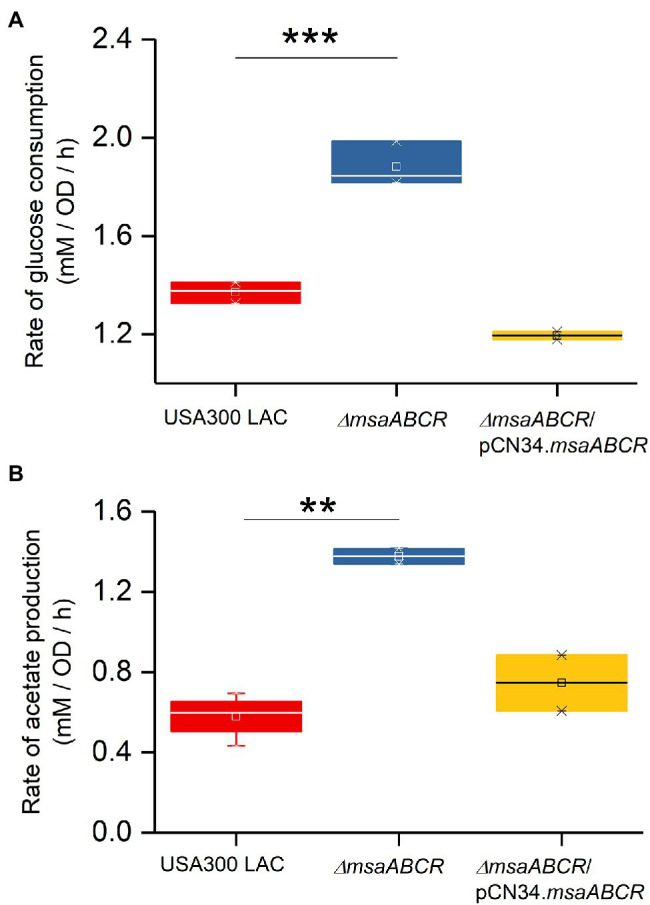
Rates of glucose consumption and acetate production when grown aerobically in TSB-50 mM glucose buffered with MOPS (50 mM, pH 7.3). The rate of glucose consumption was measured as glucose concentration (mM) consumed from 4 to 8 h of growth per OD_600_ at the 8-h time point per unit time (h; **A**). The rate of acetate production was measured as acetate concentration (mM) produced from 4 to 8 h of growth per OD_600_ at the 8-h time point per unit time (h; **B**) in the indicated *Staphylococcus aureus* strains. The box indicates Q1 and Q3 values; the solid line represents Q2. One-way ANOVA was used to compare the results for wild type with the mutant and complementation strains (^**^p < 0.005, ^***^p < 0.0005).

To assess whether the observed increases in consumption of glucose and production of acetate by the ∆*msaABCR* mutant are influenced by pH in the culture medium, we measured glucose consumption and acetate production over the first 24 h when cells were grown in TSB-50 mM glucose buffered with 50 mM MOPS (pH 7.3; Figure 5). As was observed in TSB-50 mM glucose without MOPS buffer (Figures 3C,D), after late exponential growth phase (4 h), glucose consumption and acetate production in TSB-50 mM glucose with MOPS buffer (pH 7.3) were significantly higher for the ∆*msaABCR* mutant than for the USA300 LAC (wild type) and complementation strains (Figures 5B,C). This significantly increased difference for the ∆*msaABCR* mutant is supported by the calculated rates of glucose consumption and acetate production during the period of 4–8 h of growth (Figure 6). We also confirmed a previous observation that MOPS buffer does not allow pH in the culture medium to drop below 5.5, even while all the strains were producing ~50 mM acetate (data not shown; Thomas et al., 2014). This observation suggests that acetate produced under conditions of excess glucose and buffered with MOPS produces significantly fewer neutral acetic acid molecules (as pH > 4.8) that could passively breach the *S. aureus* cell membrane and cause intracellular acidification. The improved survival of the ∆*msaABCR* mutant in TSB-50 mM glucose buffered with MOPS to a pH of 7.3 is most likely due to the inability of acetate (pKa = 4.8) to permeate cells and acidify the cytoplasm under relatively neutral conditions. In addition, we found that, under MOPS-buffered conditions, all test strains were able to transport acetate inside cells and reuse acetate after glucose was depleted from the medium, which was indicated by decreased acetate levels between the 12- and 24-h measurements (Figure 5C). Interestingly, the ∆*msaABCR* mutant reused acetate significantly faster than the USA300 LAC (wild type) and complementation strains (Figure 5C), and, as a result, the mutant strain’s growth was significantly higher at the 24-h time point (*p* < 0.0005, Figure 5A).

Overall, the ∆*msaABCR* mutant consumed glucose and produced acetate at significantly faster rates than the USA300 LAC (wild type) and complementation strains did. Therefore, we conclude that one function of the *msaABCR* operon is to repress consumption of glucose and catabolism of pyruvate to acetate during or after late exponential growth phase.

### The *msaABCR* Operon Represses Expression of the *CidR* Regulon to Repress *cidC*-Mediated, Acetate-Dependent Cell Death

**Figure 7:**
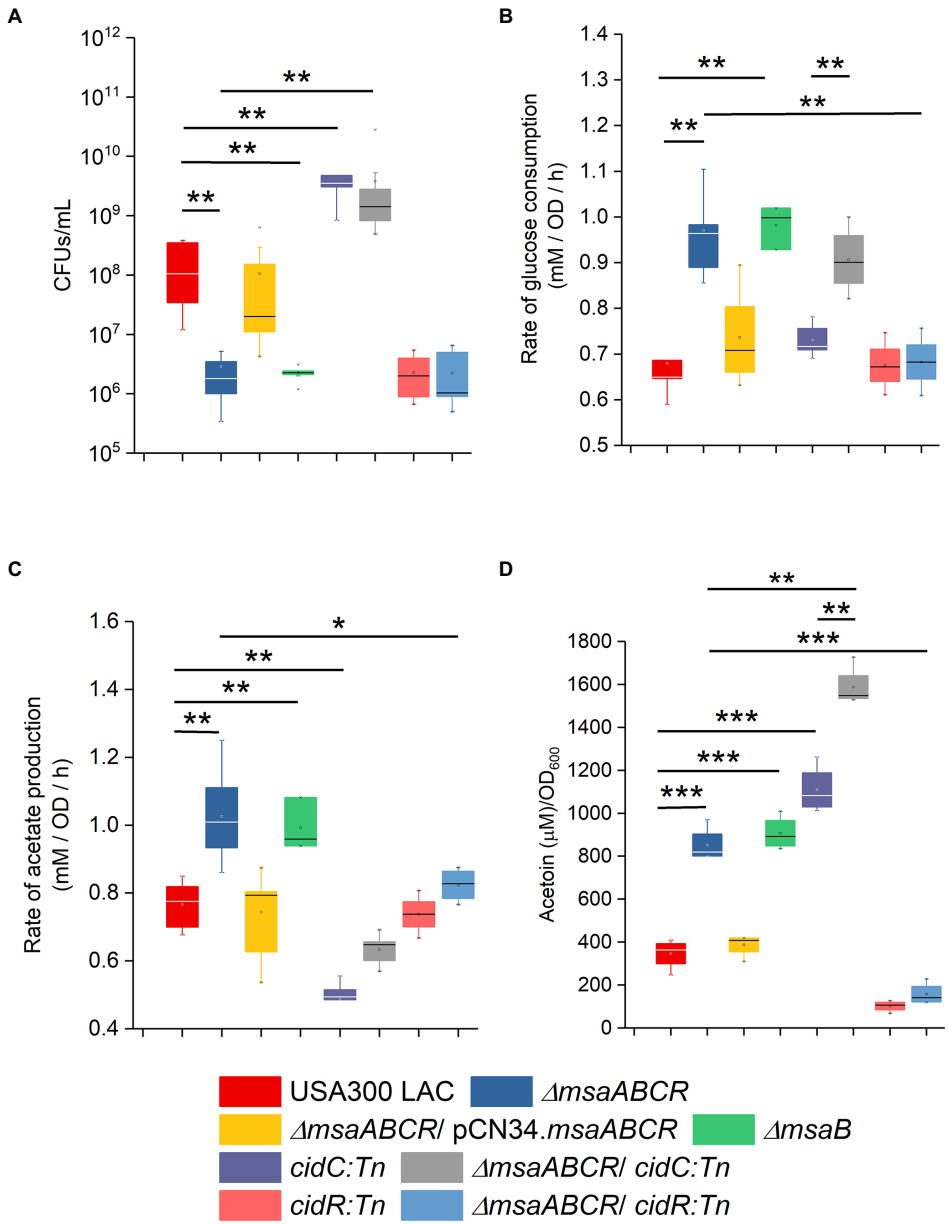
Stationary-phase survival, rate of glucose consumption, rate of acetate production, and rate of acetoin production by the indicated mutants. Cell viability of *Staphylococcus aureus* USA300 LAC (wild type), ∆*msaABCR* mutant*, ∆msaB* mutant, *cidC:Tn* mutant, ∆*msaABCR/cidC:Tn* mutant, *cidR:Tn* mutant, and ∆*msaABCR/cidR:Tn* mutant strains were monitored after 72 h of growth **(A)**. Rate of glucose consumption for all the strains was measured as the concentration of glucose (mM) consumed from 4 to 8 h of growth per OD_600_ at the 8-h time point per unit time (h; **B**). Rate of acetate production was measured as the concentration of acetate (mM) produced from 4 to 8 h of growth per OD_600_ at the 8-h time point per unit time (h; **C**). Glucose and acetate concentrations in culture supernatants were measured at the indicated growth times in TSB-50 mM glucose. Temporal accumulation of acetoin in the culture media of the indicated strains at the 12-h time point **(D)**. All the strains were grown aerobically in TSB-50 mM glucose for these experiments. The box indicates Q1 and Q3 values; the solid line represents Q2. Oneway ANOVA was used to compare the results for the two different strains (^*^p < 0.05, ^**^p < 0.005, ^***^p < 0.0005).

Several studies have reported that *cidR*, *cidABC*, *lrgAB*, *pta*–*ack*, and *alsSD* play major roles in the pathways involved in PCD and overflow metabolism (Patton et al., 2005; Yang et al., 2006; Sadykov et al., 2013, 2019; Thomas et al., 2014; Chaudhari et al., 2016; Zhang et al., 2017). Therefore, we measured with qRT-PCR the relative expression of these genes in the ∆*msaABCR* mutant cultured in TSB-50 mM glucose during late exponential growth phase (after 5 h of growth). Results showed that the relative expression of genes controlled by the *CidR* regulon (*cidR*, *cidA*, *cidC*, *lrgA*, and *alsS*) was higher in the ∆*msaABCR* mutant than in the USA300 LAC (wild type) strain (Table 1); however, expression of *pta*, *ack*, *acsA*, and *pykA* genes was not altered in the ∆*msaABCR* mutant (Table 1). These results suggest that the *msaABCR* operon represses expression of the genes of the *cidR* regulon, including those of the *cidABC*, *alsSD*, and *lrgAB* operons. When we compared expression of *CidR* regulon genes (*cidR*, *cidA*, and *alsS*) by the ∆*msaABCR* mutant with expression by the USA300 LAC (wild type) strain during late exponential growth phase in TSB without glucose, we observed a statistically insignificant increase (<2-fold) in expression of the genes but not to the same extent as when the cultures were grown in TSB-50 mM glucose (Supplementary Table 3). These results suggest that glucose or its metabolites are involved in *msaABCR*-mediated regulation of the *CidR* regulon when excess glucose is present as an environmental stressor. Among the two pathways for acetate production in *S. aureus*, the pta–ackA pathway appeared to be unaffected by deletion of the *msaABCR* operon; this was evident as no change in expression of the *pta* and *ackA* genes. Because we observed increased expression of *cidC* in the ∆*msaABCR* mutant, we examined how *cidC*-mediated acetate production is involved in survival of the ∆*msaABCR* mutant. We introduced a *cidC* transposon mutation in the ∆*msaABCR* mutant and measured survival of the ∆*msaABCR/cidC*:*Tn* double mutant during stationary phase under excess-glucose conditions (TSB-50 mM glucose; Figure 7A). Survival (assessed as CFU/ml) of the ∆*msaABCR*/*cidC:Tn* and *cidC:Tn* mutants were significantly higher than USA300 LAC (wild type; *p* < 0.0005, Figure 7A; Supplementary Figure 1A). The rate of glucose consumption of ∆*msaABCR*/*cidC:Tn* was similar to ∆*msaABCR* mutant (Figures 7B; Supplementary Figure 1B). However, the rate of acetate production was comparable to USA300 LAC but significantly less compared to ∆*msaABCR* mutant (Figures 7C; Supplementary Figure 1C). We also measured the acetoin production by all test strains (Figure 7D). We observed significant increase in acetoin production in the ∆*msaABCR* mutant (Figure 7D). These observations suggest that CidC-mediated acetate production contributed to the increased cell death in the ∆*msaABCR* mutant. Therefore, we speculated that increased expression of *cidC* contributes to increased cell death in the ∆*msaABCR* mutant.

**Table 1:** Measurement of gene expression involved in overflow metabolism in the ∆*msaABCR* mutant compared with the wild-type USA300 LAC strain.

Gene name	Relative gene expression in ∆*msaABCR* mutant in TSB-50 mM glucose
*ack*	0.813 ± 0.34
*pta*	0.847 ± 0.32
*AcsA*	1.1 ± 0.42
*pykA*	1.05 ± 0.17
*cidA*	**5.5 ± 0.58**
*cidC*	**6.9 ± 0.25**
*cidR*	**7.69 ± 0.45**
*alsS*	**2.58 ± 0.20**
*lrgA*	**3.93 ± 0.37**

Total RNA was isolated from cells grown to late exponential growth phase (5 h) in TSB-50 mM glucose. The relative fold change in gene expression was calculated by using expression of gyrB as an internal control. The values in bold represents significant changes in gene expression (>2-folds). The values reported are the mean ± standard error of the mean for at least three independent experiments.

### MsaB Regulates the CidR Regulon to Repress Overflow Metabolism

Another overflow metabolic pathway regulated by CidR is AlsSD. Because we observed increased expression of the *alsS* gene in the ∆*msaABCR* mutant (Table 1), we also assessed whether the *msaABCR* deletion affects pyruvate catabolism *via* the AlsSD pathway. For this, we measured acetoin production in cells grown in TSB-50 mM glucose for 12 h. Results showed that the ∆*msaABCR* mutant produced significantly more (~3-fold greater) acetoin in the culture medium than the USA300 LAC and complementation strains (Figure 7D). Therefore, increased expression of two metabolic genes, *cidC* and *alsS,* in the ∆*msaABCR* mutant, relative to expression in the USA300 LAC (wild type) strain, also was correlated with the respective increases in acetate (Figures 3D, 7C,) and acetoin (Figure 7D) production that were observed during the post-exponential growth phase. Furthermore, the ∆*msaABCR/cidC:Tn* mutant showed significantly higher acetoin production compared to other test strains (Figure 7D) explained why ∆*msaABCR/cidC:Tn* survived much better than other test strains (Figure 7A). All these observations suggest that the *msaABCR* operon plays an important role in repressing overflow metabolism.

MsaB is the only protein translated from the *msaABCR* operon that acts both as a transcription factor and as an RNA chaperone (Sahukhal and Elasri, 2014; Batte et al., 2016, 2018; Pandey et al., 2019); the functions of the other ncRNAs in the *msaABCR* operon remain unknown. Therefore, we next examined the contributions of the *msaB* gene in stationary-phase cell death and overflow metabolism. We confirmed the survival, the rate of glucose consumption, the rate of acetate production, and acetoin production in the ∆*msaB* mutant of USA300 LAC (Figure 7; Supplementary Figure 2). The ∆*msaB* mutant showed phenotypes similar to those of the ∆*msaABCR* mutant thus suggesting that MsaB protein is essential for these phenotypes. These observations also confirm our previous findings that ∆*msaABCR and* ∆*msaB* mutants have similar phenotypes (Sahukhal and Elasri, 2014; Batte et al., 2016, 2018; Pandey et al., 2019).

**Figure 8:**
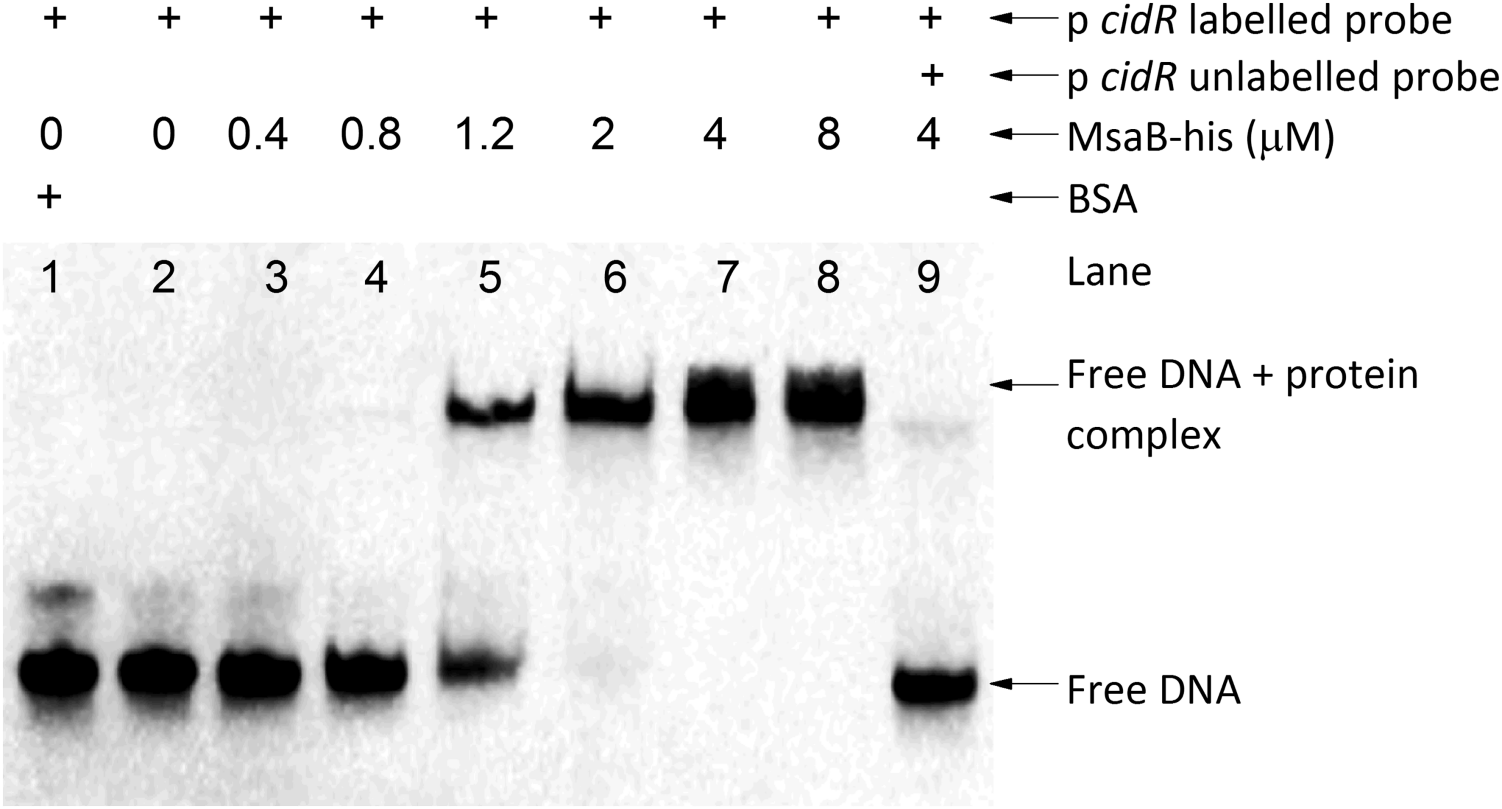
MsaB binds to the *cidR* promoter. EMSA with the *cidR* promoter. The shifted DNA–protein complex (P*cidR*–MsaB_his_ complex) is shown after an increased concentration of MsaB_his_ was incubated with a biotin-labeled *cidR* promoter target (labeled probe). The gel shift was reversed when a 100-fold excess concentration of unlabeled *cidR* promoter probe (unlabeled probe) was added to the reaction mixture, demonstrating that the label did not bind nonspecifically to MsaB_his_.

We used EMSA to investigate whether MsaB binds to the promoter region of *cidR*, *cidABC*, and *alsSD*, which are genes important for regulating pathways involved in PCD and overflow metabolism. We prepared purified MsaB protein and examined the gel shift of the protein–DNA complex with the 5′-biotinylated, amplified promoter region for each of the three target genes. The presence of a shifted band as a result of MsaB binding to the 5′-biotinylated promoter regions was observed only with the promoter region of the *cidR* gene (Figure 8) and not with the promoter regions of the *cidABC* (Supplementary Figure 3) or *alsSD* (Supplementary Figure 4) genes. Therefore, MsaB could act as a direct transcriptional regulator of the *cidR* gene. To determine the specificity of this binding, a 100-fold excess of unlabeled P*cidR* probe was added to the reaction mixture. Addition of this competitive probe resulted in an almost complete elimination of the band corresponding to the labeled P*cidR*–MsaB complex, indicating that the non-labeled specific probe competed with the labeled probe for the limited amount of MsaB (Figure 8). These results indicate that the MsaB protein binds to the *cidR* promoter and that recognition of the promoter is specific. Therefore, specific binding of MsaB protein with the *cidR* promoter and increased expression of the *cidR* gene in the ∆*msaABCR* mutant under excess-glucose conditions suggests that MsaB may be a transcriptional repressor of the *cidR* gene under these conditions.

To examine whether the cell death and overflow metabolism phenotypes observed for the ∆*msaABCR* mutant are due to MsaB affecting the *cidR* regulator, we created the ∆*msaABCR/cidR:Tn* mutant. By comparing the ∆*msaABCR/cidR:Tn* mutant with the wild-type, ∆*msaABCR* mutant, and *cidR*:*Tn* mutant strains, we assessed the following phenotypes: stationary-phase survival after 72 h of growth, glucose and acetate kinetics over 24 h, rates of glucose consumption and acetate generation after late exponential growth phase, and acetoin production after 12 h of growth (Figures 7A–D; Supplementary Figure 5). As observed in previous studies (Chaudhari et al., 2016), deletion of *cidR* caused a significant decrease in survival, relative to the wild-type strain, after 72 h of growth (Figure 7A). The ∆*msaABCR/cidR:Tn* mutant showed survival similar to that of the *cidR:Tn*-only mutant (Figure 7A). Deletion of *cidR* did not affect rates of glucose consumption and acetate production, relative to rates for the wild-type strain (Figures 7B,C; Supplementary Figure 5). However, deletion of *cidR* in the ∆*msaABCR* mutant resulted in rates of glucose consumption and acetate production that were similar to those of the wild-type and *cidR* mutant strains (Figures 7B,C). In addition, acetoin production was significantly decreased with deletion of *cidR* (Figure 7D), as shown in previous studies (Yang et al., 2006; Chaudhari et al., 2016). Deletion of *cidR* in the *∆msaABCR* mutant also significantly decreased acetoin production, relative to the ∆*msaABCR* mutant (Figure 7D), and the ∆*msaABCR/cidR:Tn* mutant produced acetoin at a level similar to that of the *cidR:Tn* mutant (Figure 7D). These results suggest that the cell death and overflow metabolism phenotypes observed in the ∆*msaABCR* mutant are due to effects of MsaB on the *cidR* regulator.

**Figure 9:**
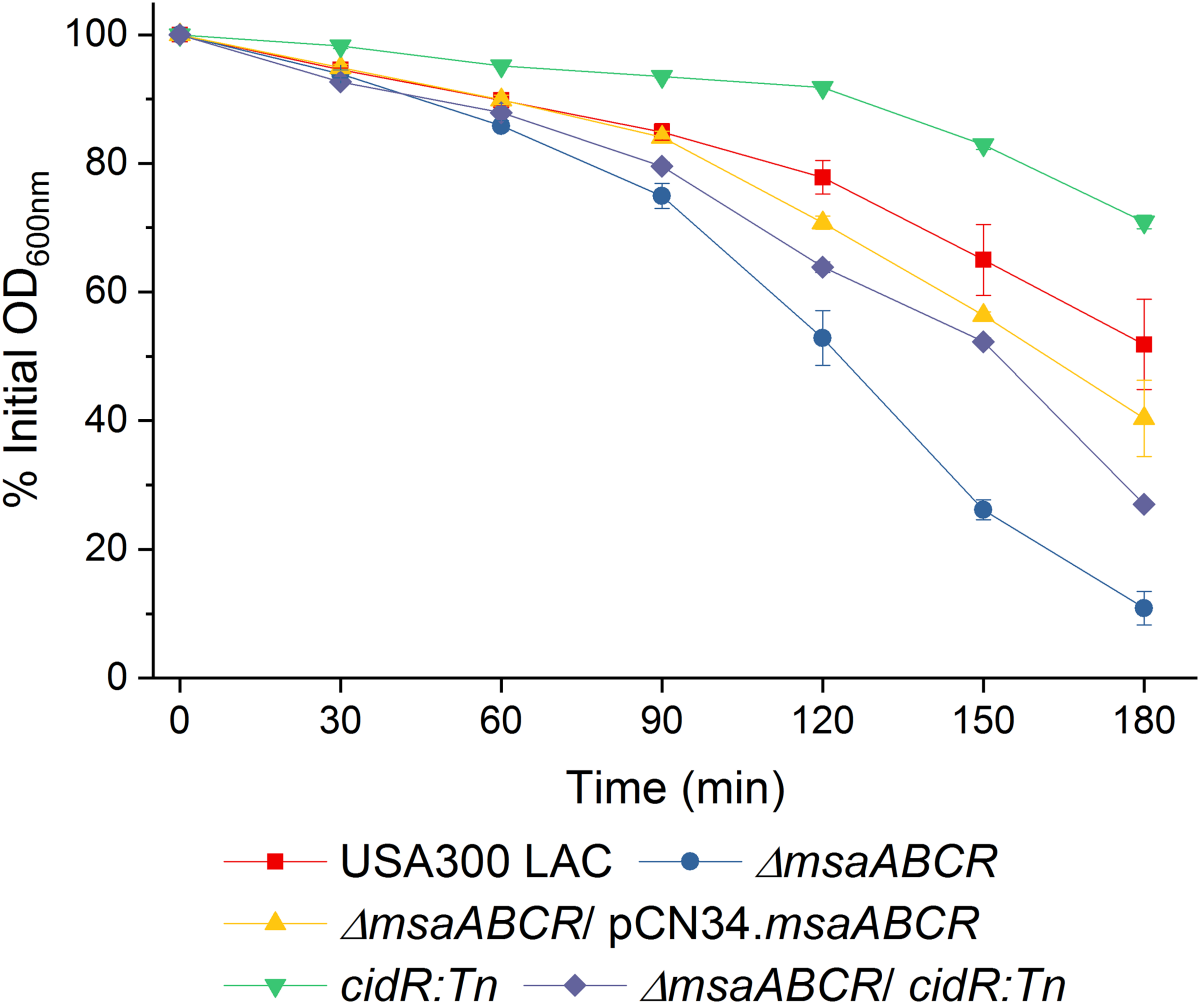
Quantitative murein hydrolase assay. Extracellular proteins (100 μg) extracted from 16-h cultures of USA300 LAC, ∆*msaABCR* mutant, *cidR:Tn* mutant, and ∆*msaABCR/cidR:Tn* mutant strains grown in TSB-50 mM glucose were each added to a 1-ml *M. luteus* cell suspension containing 1 mg/ml glucose. Murein hydrolase activity of each sample was measured as a decline in OD_600_ of the cell suspension every 30 min over 3 h. Each data point represents the mean of three independent experiments. Error bars represent the SE.

CidR increases murein hydrolase activity, *via* an unknown mechanism, when cells are grown under excess-glucose conditions (Yang et al., 2006), and the ∆*msaABCR* mutant also represses this activity (Samanta and Elasri, 2014; Sahukhal et al., 2015). To examine whether increased murein hydrolase activity in the ∆*msaABCR* mutant is due to the effects of MsaB on the *cidR* regulator, we measured this activity in the ∆*msaABCR/cidR:Tn* mutant and compared it with that of the USA300 LAC, ∆*msaABCR* mutant, and *cidR:Tn* mutant strains. The *cidR*:*Tn* mutant showed significantly decreased murein hydrolase activity, confirming the previous study (Yang et al., 2006). However, this activity in the ∆*msaABCR/cidR:Tn* mutant was not completely restored to either wild type or to the *cidR*:*Tn* mutant activity level; rather, in the ∆*msaABCR/cidR:Tn* mutant, murein hydrolase activity remained significantly lower than that of the ∆*msaABCR* mutant (Figure 9). These observations suggest that *cidR* is not the sole regulator contributing to the murein hydrolase phenotype in the ∆*msaABCR* mutant.

### Pyruvate Induces Expression of the *msaABCR* Operon to Repress Growth and Pyruvate Catabolism Under Conditions of Excess-Pyruvate During the Post-exponential Growth Phase

We observed a significant increase in expression (4.7-fold) of the *msaB* gene when the USA300 LAC (wild type) strain was grown in TSB-50 mM glucose, compared with TSB without glucose (Supplementary Table 4). We also confirmed previous reports of activation of the CidR regulon with glucose (Supplementary Table 4; Rice et al., 2005; Yang et al., 2006; Thomas et al., 2014). It has been speculated that, during overflow metabolism, when *S. aureus* cells are grown in medium with excess glucose, carbon catabolite repression may result in accumulation of intracellular pyruvate from glucose catabolism (Rice and Bayles, 2008; Sadykov and Bayles, 2012).

**Figure 10:**
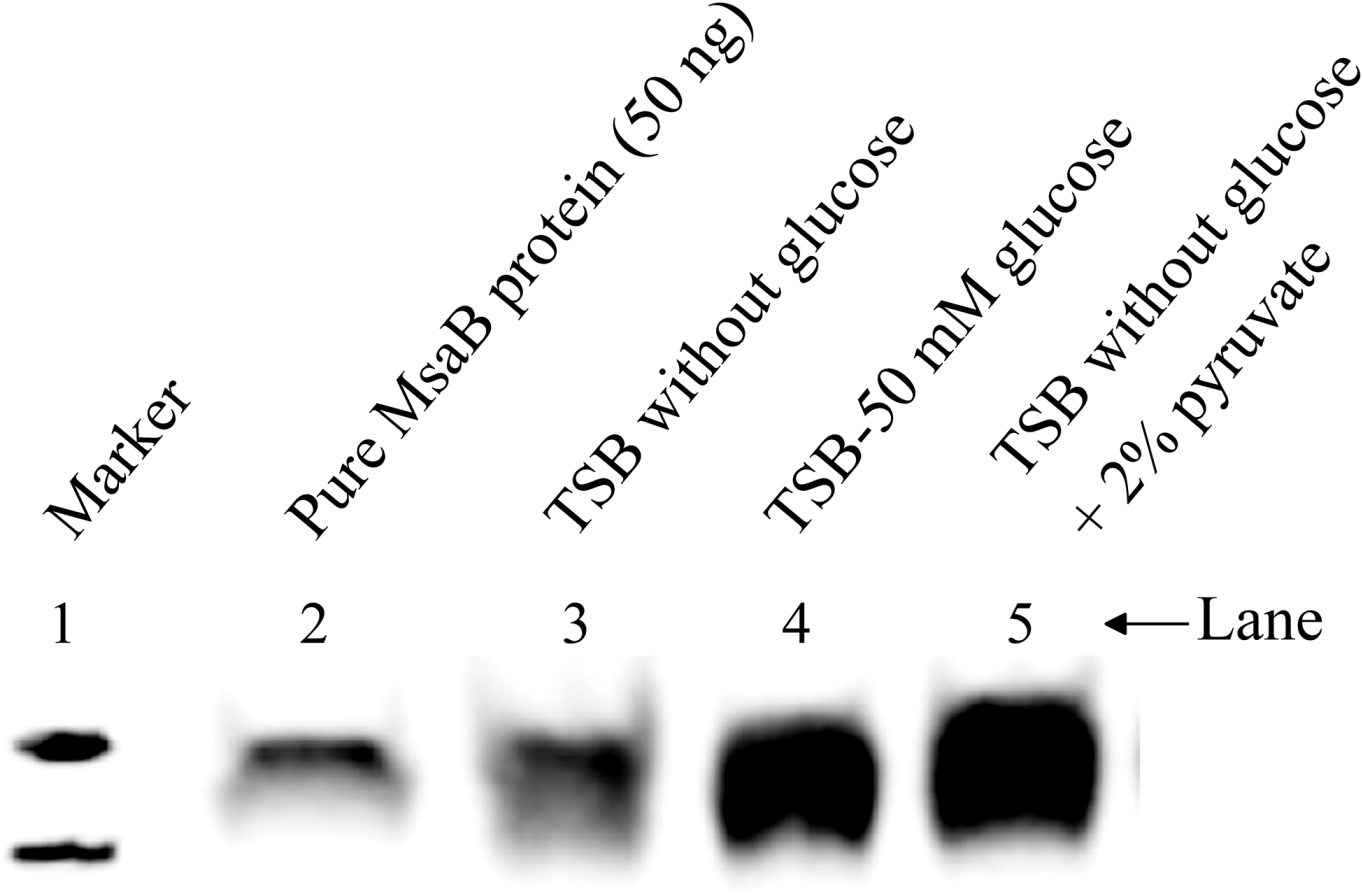
Western blot to show the MsaBhis production in USA300 LAC msaABhisCR complementation cells under different growth conditions. Cells were grown in different growth conditions like TSB without glucose, TSB-50 mM glucose, and TSB without glucose supplemented with 2% pyruvate. Cells were harvested and cell lysate were prepared. From the crude cell lysate, 25 μg of total protein was loaded in each lane. These results are representative of three independent experiments for each sample set.

To determine whether the expression of *msaABCR* that is induced by excess glucose occurs *via* its catabolism to produce excess intracellular pyruvate, we used qRT-PCR to measure the relative expression of genes in the *msaABCR* operon (*msaA*, *msaB*, *msaC*) in the USA300 LAC strain after 5 h of growth in TSB without glucose supplemented with 2% pyruvate relative to the strain grown in TSB without glucose and pyruvate (i.e., control medium). Results showed that expression of the *msaABCR* operon genes is significantly higher when grown in TSB without glucose supplemented with 2% pyruvate compared with control medium, suggesting that pyruvate induces expression of the *msaABCR* operon (Table 2). We also measured MsaB production in USA300 LAC cells grown under the following conditions: TSB without glucose (control), TSB without glucose supplemented with 2% pyruvate, and TSB-50 mM glucose. We observed that MsaB production in cells grown in TSB-50 mM glucose and in TSB without glucose supplemented with 2% pyruvate was significantly higher than in cells grown in TSB without glucose and pyruvate (Figure 10). This observation is consistent with increased expression of the *msaB* gene that occurs when cells were grown under the same conditions (Table 2 Supplementary Table 4). These results show that both glucose and pyruvate induce expression of the *msaABCR* operon and production of MsaB in the USA300 LAC strain.

**Table 2:** Expression of the *msaABCR* operon in the wild-type USA300 LAC strain in presence of supplemental pyruvate in TSB media.

Gene name	Relative expression of the *msaABCR* operon in presence of supplemental pyruvate (mean ± SDE)
*SAUSA300_1296 (msaA)*	**6.23 ± 0.51**
*SAUSA300_1295 (msaB)*	**3.59 ± 0.11**
*SAUSA300_1294 (msaC)*	**2.1 ± 0.22**

The wild-type USA300 LAC strain was grown in TSB without glucose supplemented with 2% pyruvate and TSB without glucose and pyruvate. Cells grown in TSB without glucose and pyruvate was used as control to measure the expression of msaABCR operon in the cells grown in TSB without glucose supplemented with 2% pyruvate. Total RNA was isolated from cells grown to late exponential growth phase (5 h). The relative fold change in gene expression was calculated by using expression of gyrB as an internal control. The values in bold represents significant changes in gene expression (>2-folds). The values reported are the mean ± standard error of the mean for at least three independent experiments.

**Figure 11:**
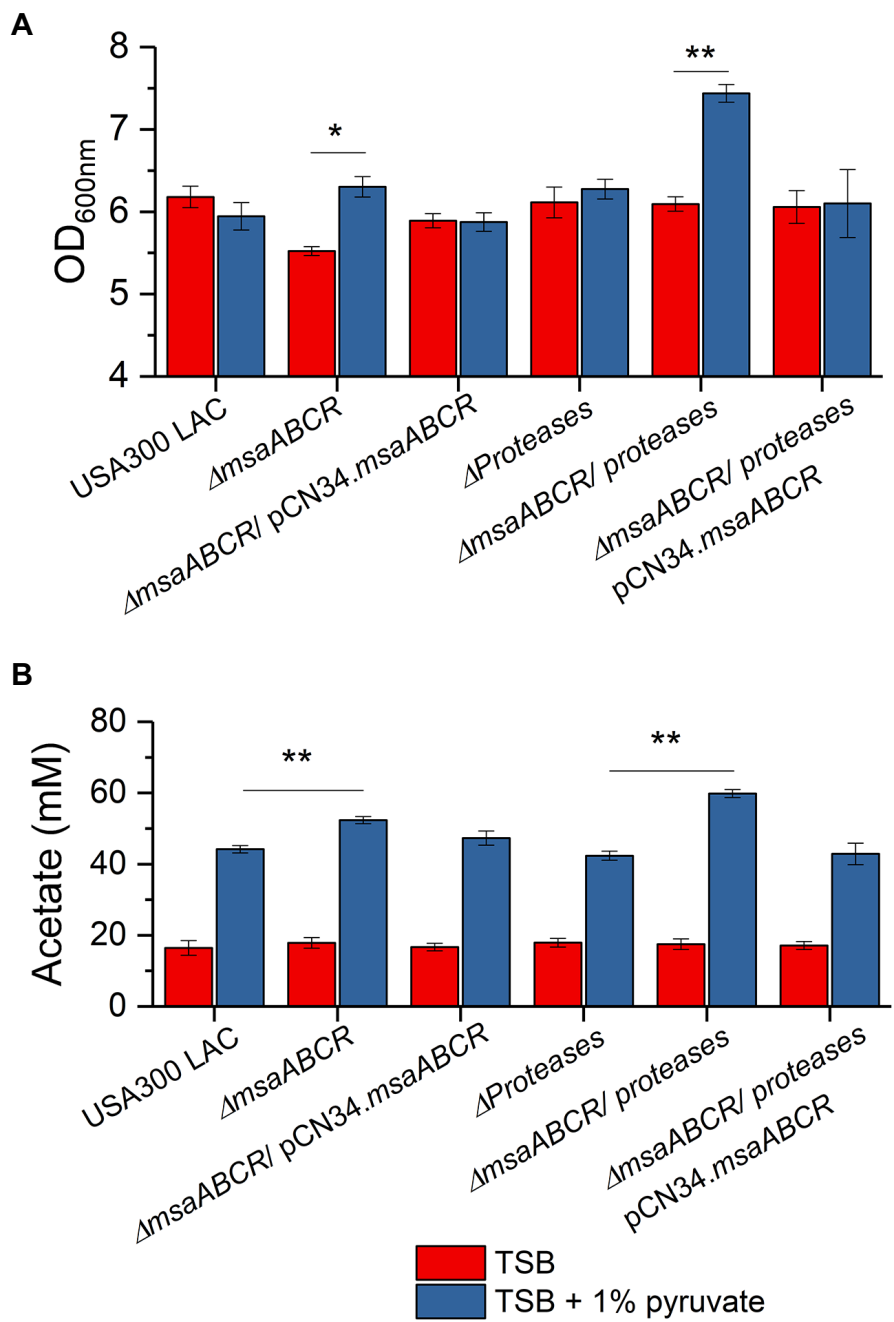
The OD_600_ measurement and acetate accumulation by the USA300 LAC (wild type), ∆*msaABCR* mutant, and complementation strains grown in TSB with 1% pyruvate. OD_600_ **(A)** and temporal accumulation of acetic acid **(B)** in the culture media of the indicated strains when grown in the indicated growth media in late exponential growth phase (5 h). Each vertical bar represents the mean of three independent experiments. Error bars represent the SE. Student’s *t*-test (unpaired) was used to compare the results for the two test groups (^*^p < 0.05, ^**^p < 0.005).

Because we observed that pyruvate induces *msaABCR* expression and MsaB production, we evaluated the ∆*msaABCR* mutant strain’s growth and ability to catabolize pyruvate to acetate when grown in TSB with 1% pyruvate for 5 h (Figure 11). For the USA300 LAC strain, growth (measured at OD_600nm_) in TSB was not statistically different from that in TSB with 1% pyruvate. Growth of the ∆*msaABCR* mutant in TSB was slightly lower than that of the USA300 LAC (wild type) strain. However, the ∆*msaABCR* mutant’s growth was significantly increased in TSB with 1% pyruvate, relative to its growth in TSB (*p* < 0.05) and were comparable to the growth of USA300 LAC under both TSB, and TSB with 1% pyruvate (Figure 11A). We also measured the amount of acetate (i.e., the product of pyruvate catabolism) produced under the same conditions. All the test strains produced same levels of acetate when grown in TSB medium. Addition of pyruvate to the medium (TSB with 1% pyruvate) invariably increased acetate production of all strains tested (Figure 11B). The ∆*msaABCR* mutant produced even more acetate than the USA300 LAC (wild type, *p* < 0.005; Figure 11B), however reversed the growth phenotype back to wild-type USA300 LAC level. Thus, pyruvate corrected the cell death phenotype without reducing acetate levels, thus suggesting that reduced fitness in the *∆msaABCR* mutant may be partly caused by depletion of pyruvate due to aberrant expression of *cidABC* and *alsSD* operons. These observations suggest that pyruvate plays a significant role in programmed cell death. However, additional investigation is required to study the role of pyruvate in cell death phenomenon.

Previously, our study showed that the ∆*msaABCR* mutant has increased protease activity, which modulates murein hydrolase activity (Sahukhal et al., 2015), and other studies have showed that increased concentrations of intracellular pyruvate induce extracellular proteases (Harper et al., 2018). In order to discern the effects of increased protease activity in the ∆*msaABCR* mutant, we used our currently tested strains combined with an all-protease-knockout strain—LAC all-protease mutant, ∆*msaABCR*/all-protease double mutant, and ∆*msaABCR*/all-protease double mutant complementation strains—and studied their growth in TSB with 1% pyruvate. We observed that growth of the ∆*msaABCR*/all-protease mutant was significantly increased in TSB with 1% pyruvate than in TSB (*p* < 0.005), but growth of the LAC all-protease mutant was the same in these two conditions (Figure 11A). In addition, when grown in TSB with 1% pyruvate, the ∆*msaABCR*/all-protease mutant produced significantly more acetate than the all-protease mutant (*p* < 0.005) and complementation strains (Figure 11B). These results show that, when cells are cultured in excess-pyruvate conditions, induces production of MsaB. Increased MsaB thus might have important role in repressing expression of *cidABC* and *alsSD* operons *via cidC* under increased intracellular pyruvate condition.

## Discussion

In this study, we investigated the role of the *msaABCR* operon in regulation of the CidR regulon, and we defined the mechanism of cell death as regulated by the *msaABCR* operon in the USA300 LAC strain. The results showed that the ∆*msaABCR* mutant has increased CidC-mediated, weak-acid–dependent cell death. We observed an increased rate of glucose consumption and of acetate and acetoin production in the ∆*msaABCR* mutant, relative to the USA300 LAC (wild type) strain, under excess-glucose conditions. We showed that *msaABCR* represses expression of the *cidABC* and *alsSD* operons, which repress pyruvate catabolism to acetate and acetoin, respectively. Our results suggest that MsaB represses overflow metabolism *via cidR* by binding to its promoter region. Pyruvate also was shown to induce expression of the *msaABCR* operon. Our findings further clarify the role of the *msaABCR* operon in staphylococcal metabolic adaption under excess glucose condition.

Previous studies have shown that addition of glucose or antibiotics, such as vancomycin, induces biofilm formation in *S. aureus* (Ferreira et al., 2012; You et al., 2014; Hsu et al., 2015; He et al., 2017). Consistent with previous studies, we observed induced biofilm formation in the USA300 LAC (wild type) strain with excess glucose and/or subinhibitory concentrations of vancomycin (Hsu et al., 2011, 2015; Mirani and Jamil, 2011; He et al., 2017). However, these conditions failed to induce biofilm formation in the ∆*msaABCR* mutant (Figure 1), indicating that the *msaABCR* operon plays a role in vancomycin- and glucose-induced biofilm formation. Few studies have shown that *cidA* mediates induction of biofilm development under these conditions (Hsu et al., 2011, 2015). In addition, release of eDNA *via cidA*-mediated cell death is a major contributor to vancomycin-enhanced biofilm formation (Hsu et al., 2011, 2015; Mirani and Jamil, 2011). Defective biofilm development in the ∆*msaABCR* mutant under normal conditions and conditions of excess glucose or subinhibitory antibiotic stress, as well as our previous report of its reduced biofilm formation in bone implants in a rat osteomyelitis model, suggest that the *msaABCR* operon is essential for establishing staphylococcal biofilm infections (Sahukhal et al., 2015, 2020). Previously, we showed that deletion of the *msaABCR* operon results in a defective biofilm phenotype, with significantly increased dead cell biomass, decreased live cell biomass, and decreased biofilm thickness (Sahukhal and Elasri, 2014; Sahukhal et al., 2015). The process of cell death is essential for adequate biofilm formation, but proper biofilm development requires maintenance of a homeostatic balance between the dying and growing cells (Bayles, 2007; Rice and Bayles, 2008; Sadykov and Bayles, 2012; Thomas et al., 2014; Windham et al., 2016). Thus, we hypothesized that the *msaABCR* operon plays an important role in maintaining a balance between cell death and cell growth during normal, excess glucose and vancomycin induced biofilm development. It has been shown that the expression dynamics of the major genes involved in cell death, such as *cidR*, *cidABC*, and *alsSD*, that are observed in staphylococcal biofilm microcolonies are mimicked during planktonic growth in TSB-50 mM glucose (Thomas et al., 2014). Therefore, to better understand the mechanism of programmed cell death during biofilm formation, stationary-phase survival (Figure 2), overflow metabolism (Figures 3–7), and transcription of genes regulated by the CidR regulon (Table 1) by *msaABCR* operon, we performed these experiments in TSB-50 mM glucose.

Previous studies have proposed that acetic acid (produced during glucose catabolism) accumulates in biofilm microcolonies, creating acidic microenvironments due to low diffusion within the biofilm structure. In an environment with a pH close to the pKa of acetate (~4.8), acetate is protonated to its neutral acetic acid form, which can easily diffuse through a subpopulation of cells in microcolonies. Increased localized accumulation of acetic acid within these microcolonies then increases intracellular acidification, which can lead to unfolding or misfolding of proteins and reduce the functionality of the electron transport chain, which can, in turn, result in increased production of reactive oxygen species (ROS) and, ultimately, cause cell death in *S. aureus* (Bayles, 2007; Sadykov and Bayles, 2012; Thomas et al., 2014; Chaudhari et al., 2016). The present study showed that the ∆*msaABCR* mutant consumes glucose and produces acetate at significantly higher rates than the wild-type USA300 LAC strain during overflow metabolism (Figures 3, 4), so we hypothesized that increased acetate production and decreased extracellular pH increases cell death in the *∆msaABCR* mutant. This hypothesis is supported by our observation that under MOPS-buffered condition, the increased cell death phenotype of Δ*msaABCR* mutant reverted to wild-type level without affecting the rate of glucose consumption and acetate production (Figures 5B,C). The MOPS buffer does not allow pH in the culture medium to drop below 5.5, despite the Δ*msaABCR* mutant produced ~50 mM acetate at 12-h time point. Under MOPS-buffered conditions, all test strains were able to reutilize acetate as secondary source of carbon after glucose was depleted from the medium (Figure 5C). Interestingly, the Δ*msaABCR* mutant reutilized acetate significantly faster than the USA300 LAC (wild type) and complementation strains (Figure 5C). In our previous study, we showed that *msaABCR* operon represses TCA cycle (Pandey et al., 2021). Increased reutilization of acetate in Δ*msaABCR* mutant is most likely due to increased TCA activity. However, further studies are needed to investigate the role of *msaABCR* operon in acetate reutilization mechanism.

The ∆*msaABCR/cidC:Tn* mutant produced a significantly lower amount of acetate than the ∆*msaABCR* mutant. Stationary-phase survival of the ∆*msaABCR* mutant also was reversed to the USA300 LAC (wild type) level when we introduced the *cidC* mutation into this mutant (Figure 7). These observations also suggest that increased acetate production *via* the CidC pathway contributes to increased weak-acid-dependent cell death in the ∆*msaABCR* mutant. Previously, our lab found the ∆*msaABCR* mutant to be defective in its oxidative stress response (Pandey et al., 2019). Therefore, we hypothesized that the inability of the ∆*msaABCR* mutant to cope with acetate-mediated ROS oxidative stress may also contribute to increased cell death in this mutant. Interestingly, mutation of *cidC* in the *∆msaABCR* mutant reduced acetate production but did not reduce the rate of glucose consumption compared with the ∆*msaABCR* mutant without mutation (Figure 7B). However, the ∆*msaABCR*/*cidC:Tn* double mutant produced a significantly greater amount of acetoin than the *cidC* mutant. This suggests that the excess pyruvate (resulting from excess glucose consumption) was funneled through the AlsSD pathway to produce acetoin when acetate formation *via* the CidC pathway was blocked (by mutation of the *cidC* gene) in the ∆*msaABCR/cidC:Tn* mutant (Figure 7D). This finding indicates a further role for the *msaABCR* operon in repression of pyruvate catabolism and maintenance of pyruvate homeostasis.

In this study, we also showed that MsaB binds to the *cidR* gene promoter region, suggesting that it act as a transcriptional regulator of *cidR* (Figure 8). CidR is a transcriptional activator of two operons, *cidABC* and *alsSD,* that display pro- and anti-death functions, respectively (Yang et al., 2006; Thomas et al., 2014; Chaudhari et al., 2016; Sadykov et al., 2019). These two operons have been shown to play important roles in catabolism of pyruvate. The *alsSD* operon encodes acetolactate synthase and acetolactate decarboxylase, which are required for converting pyruvate to the neutral byproduct acetoin, whereas *cidC* encodes pyruvate:menaquinone oxidoreductase for decarboxylation of pyruvate to form acetate (Yang et al., 2006; Thomas et al., 2014; Chaudhari et al., 2016; Sadykov et al., 2019). Several studies suggest that the metabolic activities of the *alsSD* and *cidC* gene products play important roles in determining the direction of carbon flux at the pyruvate node, thereby determining cell fate during aerobic growth in excess glucose (Thomas et al., 2014; Chaudhari et al., 2016; Sadykov et al., 2019). The *cidABC* operon previously was shown to be transcribed into two different overlapping transcripts, *cidABC* and *cidBC,* depending on growth conditions (Rice et al., 2004). Sigma factor B (SigB) was found to activate the smaller *cidBC* transcript, produced during exponential growth phase (Rice et al., 2004), while the LTTR family transcriptional regulator, CidR, induces the full-length *cidABC* transcript during late exponential growth phase after acidification of the culture medium occurs due to increased acetate when cells are grown under excess-glucose conditions (Yang et al., 2006; Chaudhari et al., 2016). In our study, deletion of *msaABCR* resulted in increased *cidR* expression, increased *cidABC* and *alsSD* transcription, and increased acetate and acetoin production during the post-exponential phase. However, expression of the *pta* and *ackA* genes was not altered in the ∆*msaABCR* mutant (Table 1). Previous studies have reported that deletion of the *msaABCR* operon results in decreased *sigB* expression during exponential growth phase (Sahukhal and Elasri, 2014). All these results suggest that increased acetate and acetoin production in the ∆*msaABCR* mutant most likely is due to increased *cidR* expression that leads to the aberrant expression of *cidABC* and *alsSD*. Interestingly, deletion of *cidR* in the ∆*msaABCR* mutant reversed the rate of glucose consumption, acetate and acetoin production, and stationary-phase survival comparable to wild type and/or *cidR* mutant strains (Figures 7A–D; Supplementary Figure 5). These observations suggest that the observed cell death and overflow metabolism phenotypes in the ∆*msaABCR* mutant occur *via* the effects of MsaB on the *cidR* regulator.

Studies have shown that an acidic pH within microcolonies can activate the CidR regulon in a subpopulation of cells (Thomas et al., 2014). CidR activation causes increased murein hydrolase activity and *cidABC* activation, leading to a feed-forward loop in which toxic levels of acetate are reached as a result of increased CidC activity, ultimately resulting in cell death with the exhaustion of repair mechanisms (Patton et al., 2005; Thomas et al., 2014; Chaudhari et al., 2016; Marshall et al., 2016). Thus, regulation of *cidR* expression plays an important role in determining the fate of staphylococcal cells. Our results suggest that the *msaABCR* operon, through MsaB binding to the *cidR* promoter (Figure 8), is involved in controlling *cidR* expression to limit autolysis and acetate-dependent potentiation of cell death.

In our previous study, we showed that the ∆*msaABCR* mutant has increased expression of cell-wall–bound and extracellular murein hydrolases, and this activity was further increased by enhanced activity of extracellular proteases (Sahukhal et al., 2015; GC et al., 2019). We also showed that deletion of all proteases in the ∆*msaABCR* mutant decreased dead cells in biofilms of the ∆*msaABCR* mutant strain (Sahukhal et al., 2015; GC et al., 2019). CidR has been found to activate murein hydrolase activity, *via* an unknown mechanism, in cells grown in excess-glucose conditions (Yang et al., 2006). This activity decreased as a result of deletion of *cidR* in the *∆msaABCR* mutant, relative to activity in the ∆*msaABCR* mutant (Figure 9). However, the ∆*msaABCR/cidR:Tn* mutant had greater murein hydrolase activity than the *cidR:Tn*-only mutant (Figure 9). These observations suggest that CidR-mediated murein hydrolase activity partially contributes to the increased activity in the ∆*msaABCR* mutant and suggest the existence of another mechanism that involves the *msaABCR* operon in repression of this activity.

During overflow metabolism, repression of carbon catabolites may result in the accumulation of intracellular pyruvate (Rice and Bayles, 2008; Sadykov and Bayles, 2012). Several studies have speculated that pyruvate is an inducer molecule for activation of *cidR* expression when cells are grown in excess glucose under acidic conditions (Thomas et al., 2014; Sadykov et al., 2019). Thus, pyruvate is the central metabolite directing overflow metabolism. Because pyruvate lies at the junction of several essential pathways in *S. aureus* cells, tight control of pyruvate homeostasis and its fate is crucial for metabolic adaptability in *S. aureus*. In this study, we showed that pyruvate induces expression of the *msaABCR* operon and subsequent production of MsaB. We also showed that MsaB indirectly represses pathways of pyruvate catabolismby repressing CidR regulon (*cidC* and *alsSD) via* MsaB binding to the *cidR* promoter. Therefore, these results suggest that the *msaABCR* operon (MsaB) might respond to intracellular pyruvate concentration, which would allow it to direct optimal expression of *cidR* to regulate programmed cell death during biofilm development and pyruvate catabolism, thereby maintaining pyruvate homeostasis. However, further studies are needed to determine whether MsaB senses pyruvate directly or indirectly. Several transcriptional regulators, including AgrAC, SaeRS, and ArlRS, have been found to be essential for pyruvate-mediated virulence regulation (Harper et al., 2018). Thus, we also cannot rule out the involvement of other transcriptional regulators and systems that are controlled by changes in glycolytic flux in pyruvate sensing by MsaB protein. Therefore, a detailed analysis of intracellular pyruvate sensing by MsaB requires further study.

**Figure 12:**
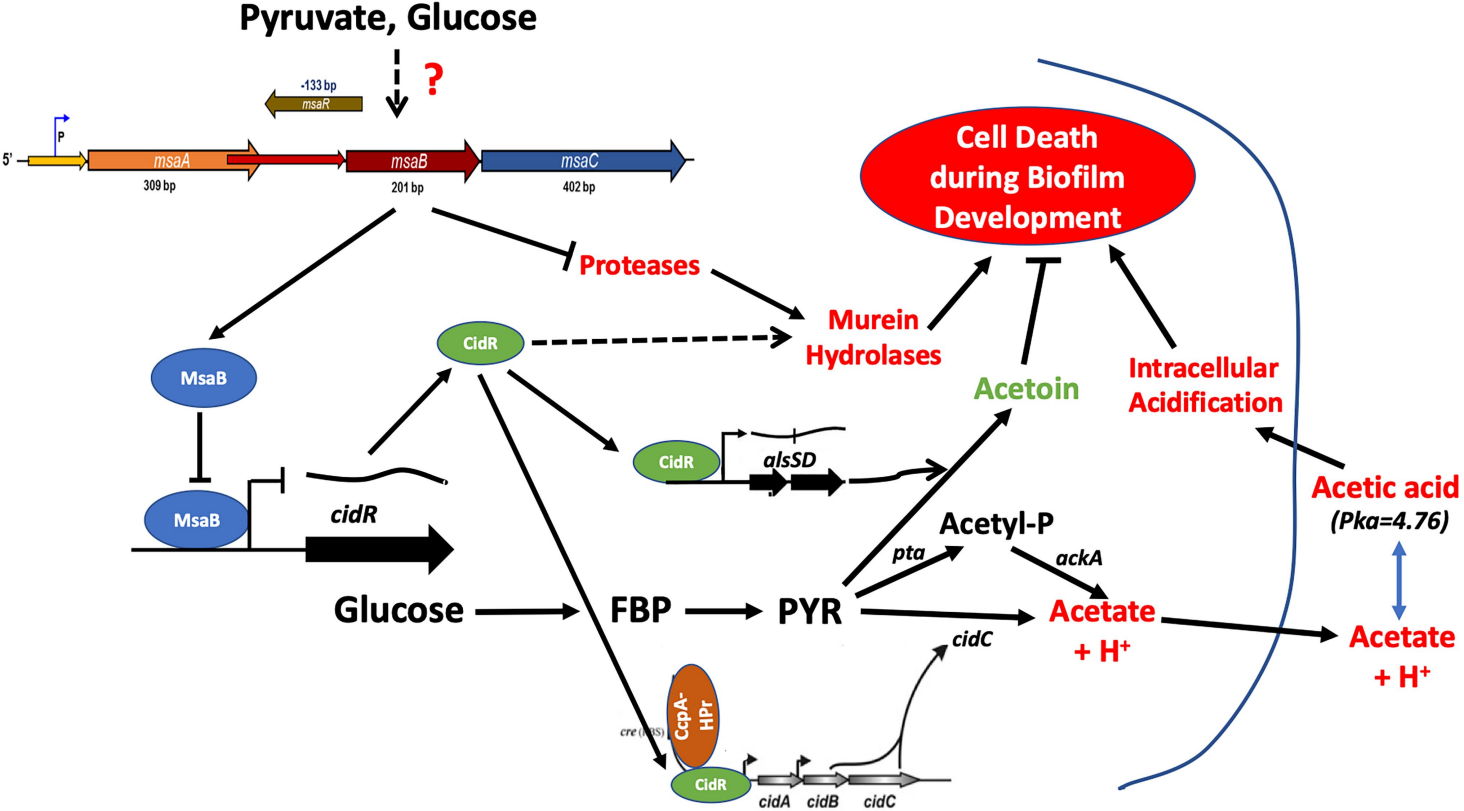
MsaB regulatory pathway during staphylococcal overflow metabolism. Pyruvate induces MsaB production, which, in turn, represses pyruvate catabolic pathways *via* the *cidC* and *alsSD* pathways by repressing CidR activity.

In conclusion, this report suggests that the *msaABCR* operon regulates the process of weak-acid-dependent cell death in *S. aureus* (Figure 12). This study also showed that the *msaABCR* operon directly represses the *cidR* regulon to repress pyruvate catabolism during overflow metabolism. Our new findings help decipher the role of the *msaABCR* operon in staphylococcal overflow metabolic adaption during biofilm development, as well as its roles in pyruvate catabolism and maintenance of pyruvate homeostasis (Figure 12). Going forward, we will seek to further define the mechanism by which the *msaABCR* operon senses the central metabolite, pyruvate. Continued research is crucial for understanding *S. aureus* metabolic adaptation during chronic infection and for developing alternative treatments to combat the current rise in highly virulent, antibiotic-resistant strains in clinical infections.

## Data Availability Statement

The datasets presented in this study can be found in online repositories. The names of the repository/repositories and accession number(s) can be found in the article/Supplementary Material.

## Author Contributions

GSS and MOE supervised the project. BGC performed the experiments. All authors designed the project, wrote the manuscript, and read and approved the final manuscript.

## Funding

This work was partially supported by the Mississippi INBRE, funded by an Institutional Development Award (IDeA) from the National Institute of General Medical Sciences of the National Institutes of Health under grant number P20GM103476 and by startup funding (Z1-50328-01) from the University of Arkansas for Medical Sciences.

## Conflict of Interest

The authors declare that the research was conducted in the absence of any commercial or financial relationships that could be construed as a potential conflict of interest.

## Publisher’s Note

All claims expressed in this article are solely those of the authors and do not necessarily represent those of their affiliated organizations, or those of the publisher, the editors and the reviewers. Any product that may be evaluated in this article, or claim that may be made by its manufacturer, is not guaranteed or endorsed by the publisher.
