# Supplementary material for: Delineating the Role of the msaABCR Operon in Staphylococcal Overflow Metabolism
Source: Front Microbiol. 2022 Jun 3;13:914512. doi: 10.3389/fmicb.2022.914512 (PMC9204165; doi:10.3389/fmicb.2022.914512)
Supplement: Supplementary file 1 [file Data_Sheet_1.docx]

**SUPPLEMENTARY FIGURES:**

**

**

**Supplementary Figure 1. Glucose and acetate kinetics of the *cidC:Tn* and ∆*msaABCR/cidC:Tn* mutants in TSB-50 mM glucose.** OD_600nm_ values of the culture medium were determined at the indicated times (**A**). Temporal depletion of glucose from (**B**) or accumulation of acetic acid in (**C**) the culture media of the indicated strains. Glucose and acetate concentrations in culture supernatants were measured at the indicated growth times in TSB-50 mM glucose by using a commercially available kit (R-Biopharm). Each data point represents the mean of three independent experiments. Error bars represent the standard error.

**

**

**Supplementary Figure 2. Growth curve and glucose and acetate kinetics of the ∆*msaABCR* mutant and ∆*msaB* mutant in TSB-50 mM glucose.** OD_600nm_ values of the culture medium were determined at the indicated times (**A**). Temporal depletion of glucose from (**B**) or accumulation of acetic acid in (**C**) the culture media of the indicated strains. Glucose and acetate concentrations in culture supernatants were measured at the indicated growth times in TSB plus 35 mM glucose by using a commercially available kit (R-Biopharm). Each data point represents the mean of three independent experiments. Error bars represent the standard error.

**

**

**Supplementary Figure 3. Electrophoretic mobility shift assay (EMSA) with the *cidABC* promoters.** No shifted DNA–protein complex (P*cidABC*–MsaB_his_ complex) occurred when an increased concentration of MsaB_his_ was incubated with biotin-labeled *cidABC* promoter targets (labeled probes).

**

**

**Supplementary Figure 4. Electrophoretic mobility shift assay (EMSA) with the *alsSD* promoters.** No shifted DNA–protein complex (P*alsSD*–MsaB_his_ complex) occurred when an increased concentration of MsaB_his_ was incubated with biotin-labeled *alsSD* promoter targets (labeled probes).

**

**

**Supplementary Figure 5. Growth curve and glucose and acetate kinetics of the *cidR* and ∆*msaABCR/cidR:Tn* mutants in TSB-50 mM glucose.** OD_600nm_ values of the culture medium were determined at the indicated times (**A**). Temporal depletion of glucose from (**B**) or accumulation of acetic acid in (**C**) the culture media of the indicated strains. Glucose and acetate concentrations in culture supernatants were measured at the indicated growth times in TSB-50 mM glucose by using a commercially available kit (R-Biopharm). Each data point represents the mean of three independent experiments. Error bars represent the standard error.
