# Supplementary material for: Delineating the Role of the msaABCR Operon in Staphylococcal Overflow Metabolism
Source: Front Microbiol. 2022 Jun 3;13:914512. doi: 10.3389/fmicb.2022.914512 (PMC9204165; doi:10.3389/fmicb.2022.914512)
Supplement: Supplementary file 2 [file Data_Sheet_2.docx]

**SUPPLEMENTARY TABLES:**

**Supplementary Table 1. Strains used in the study.**

| **Strains or plasmids** | **Relevant characteristics** | **Reference/ source** |  |
| --- | --- | --- | --- |
| *E. coli* DH5α | | F^−^ ϕ80lacZΔM15 recA1 | Life Technologies |
| RN4220 | | Restriction-deficient laboratory strain | NARSA |
| USA300 LAC | | CA-MRSA USA300 strain | Dr. Lindsey Shaw |
| USA300 LAC ∆*msaABCR* | | USA300 LAC *msaABCR*-deletion mutant | (Sahukhal and Elasri, 2014) |
| USA300 LAC ∆*msaB* | | USA300 LAC *msaB*-deletion mutant | (Elbarasi, 2014) |
| USA300 LAC ∆*msaABCR.*  *pCN34.msaABCR* | | (pCN34–*msaABCR* operon) complementation into USA300 LAC *msaABCR*-deletion mutant | (Sahukhal and Elasri, 2014) |
| USA300 LAC *msaAB_his_CR* complementation | | (pCN34–*msaAB_his_CR* operon) complementation with His-tagged *msaB* into USA300 LAC ∆*msaABCR* | This study |
| MOE 458 | | USA300 LAC proteases deletion mutant | Dr. Mark Smeltzer |
| MOE 466 | | USA300 LAC ∆*msaABCR* & proteases mutant | (Sahukhal et al., 2015) |
| USA300 LAC *CidC:Tn* | | USA300 LAC *cidC* transposon mutant | This study |
| USA300 LAC ∆*msaABCR*/*CidC:Tn* | | USA300 LAC ∆*msaABCR* and *cidC* transposon mutant | This study |
| USA300 LAC *CidR:Tn* | | USA300 LAC *cidR* transposon mutant | This study |
| USA300 LAC ∆*msaABCR*/*CidR:Tn* | | USA300 LAC ∆*msaABCR* and *cidR* transposon mutant | This study |

**Supplementary Table 2. Primers used in the study.**

| **Primer** | **Sequence** (**5′ to 3′**) | **Reference** |
| --- | --- | --- |
| Primers for real-time PCR | | |
| RT *gyrB* F | GGTGCTGGGCAAATACAAGT | (Sahukhal et al., 2015) |
| RT *gyrB* R | TCCCACACTAAATGGTGCAA | (Sahukhal et al., 2015) |
| RT *ackA* F | ATGTTATCGTATTTACAGCAGG | This study |
| RT *ackA* R | CCAGTAAACACCCATAAATTCT | This study |
| RT *pta* F | GATCCAAAAGTTGCAATGTTAA | This study |
| RT *pta* R | TTTTGTTGTGCTAATTTGACAG | This study |
| RT *acsA* F | TATATTTTGCGTTGTTAGGTGT | This study |
| RT *acsA* R | AGCTTCACTGTTCTCTAATCTA | This study |
| RT *pykA* F | AGTACAGATGCATTGTTAAACA | This study |
| RT *pykA* R | GTACACCAGCAGTAATAATGAT | This study |
| RT *cidA* F | GGGTAGAAGACGGTGCAAAC | This study |
| RT *cidA* R | TTTAGCGTAATTTCGGAAGCA | This study |
| RT *cidC* F | TATTTGTCCTAACGACTTATTAACTG | This study |
| RT *cidC* R | GTCTTTATATTTTGGTGATACTACTGTT | This study |
| RT *cidR* F | CGCCCTTATTTGATAGAAGTAAAA | This study |
| RT *cidR* R | GATGGTAAATAATCATACAGTGCA | This study |
| RT *alsS* F | TTGGATGGCACGTAATTTCA | This study |
| RT *alsS* R | GCCAGCAACGGATACAACTT | This study |
| RT *lrgA* F | GCATCAAAACCAGCACACTTT | This study |
| RT *lrgA* R | TGATGCAGGCATAGGAATTG | This study |
| RT *msaC* F | CCAGAAATCATTATCGGAATCACTA | (Sahukhal and Elasri, 2014) |
| RT *msaC* R | TTAGTTTTCGGTGTATTATCTGCAA | (Sahukhal and Elasri, 2014) |
| RT *msaB* F | TTTATCGAAGTTGAAGGAGAAAATG | (Sahukhal and Elasri, 2014) |
| RT *msaB* R | ACTCAACAGCTTGACCTTCTTCTAA | (Sahukhal and Elasri, 2014) |
| RT *msaA* F | TCGATAACTATGTCACAGGCAAATA | (Sahukhal and Elasri, 2014) |
| RT *msaA* R | TTGTAAATCCTCTTCACAATCTTCG | (Sahukhal and Elasri, 2014) |
| Primers for EMSA | |  |
| P*cidABC*-EMSA | AGTGAAATTTAGAGAGCGTTTCCATAGAAAATAGTAATACAAACC ATAAAAAAAGAGTAT | (Sadykov et al., 2019) |
| P*alsSD*-EMSA | ATACTCTTTTTTTATGGTTTGTATTACTATTTTCTATGGAAACGCTC TCTAAATTTCACT | (Sadykov et al., 2019) |
| P*cidR*-EMSA F | Biotin-ACTTGAGCCATTAATATAATACCA | This study |
| P*cidR*-EMSA R | TCCACTAACATCATCCTAACTATT-Biotin | This study |
| Primers for verification of transposon mutant | | |
| *CidC* Tn F | GACAGAAAGGGAGGCTATTA | This study |
| *CidC* Tn R | CATTACCAGTAACACGCAAG | This study |
| *CidR* Tn F | CTCAATACCTCACCTCGATT | This study |
| *CidR* Tn R | CCAAACAACACCTAATTCCC | This study |

**Supplementary Table 3**. **Expression of genes involved in the *cidR* regulon in the ∆*msaABCR* mutant relative to the USA300 LAC** (**wild type**) **strain grown in TSB without glucose**.

| **Gene name** | **Relative expression in the ∆*msaABCR* mutant in TSB without glucose** |
| --- | --- |
| *cidA* | **1.73 ± 0.25** |
| *cidR* | **1.82 ± 0.20** |
| *alsS* | **1.65 ± 0.14** |

**Supplementary Table 3. Fold change measurement of genes of the *cidR* regulon in the ∆*msaABCR* mutant compared with the USA300 LAC (wild type) strain.** Total RNA was isolated from cells grown in TSB without glucose or in TSB-50 mM glucose in the late exponential growth phase (5 h). The relative fold change in gene expression was calculated by using *gyrB* as the internal control. Each value is the mean ± standard error of the mean for at least three independent experiments.

**Supplementary Table 4.** **Expression of the *cidR* regulon in the USA300 LAC (wild type) strain when grown in the presence of excess glucose.**

| **Gene name** | **Relative expression in TSB-50 mM glucose relative to TSB without glucose**  **(mean ± SDE)** |
| --- | --- |
| ***cidA*** | **7.28 ± 1.23** |
| ***cidR*** | **2.39 ± 0.31** |
| ***alsS*** | **5.95 ± 0.73** |
| ***msaB*** | **4.71 ± 0.32** |

**Supplementary Table 4**. **Fold-change in expression of the *msaABCR* operon in the USA300 LAC strain when grown in TSB-50 mM glucose, relative to TSB without glucose.** Total RNA was isolated (RNeasy RNA extraction kit, Qiagen) from cells grown to late exponential growth phase (5 h). The relative fold change in gene expression was calculated by using *gyrB* as the internal control. Each value is the mean ± standard error of the mean for at least three independent experiments.

**REFERENCES**

ELBARASI, A. 2014. Identification and characterization of msaB gene involved in biofilm formation and virulence in Staphylococcus aureus.

SADYKOV, M. R., WINDHAM, I. H., WIDHELM, T. J., YAJJALA, V. K., WATSON, S. M., ENDRES, J. L., BAVARI, A. I., THOMAS, V. C., BOSE, J. L. & BAYLES, K. W. 2019. CidR and CcpA synergistically regulate Staphylococcus aureus cidABC expression. *Journal of Bacteriology***,** JB.00371-19.

SAHUKHAL, G. S., BATTE, J. L. & ELASRI, M. O. 2015. msaABCR operon positively regulates biofilm development by repressing proteases and autolysis in Staphylococcus aureus. *FEMS Microbiol Lett,* 362.

SAHUKHAL, G. S. & ELASRI, M. O. 2014. Identification and characterization of an operon, msaABCR, that controls virulence and biofilm development in Staphylococcus aureus. *BMC Microbiol,* 14**,** 154.
